# Stim and Orai mediate constitutive Ca^2+^ entry and control endoplasmic reticulum Ca^2+^ refilling in primary cultures of colorectal carcinoma cells

**DOI:** 10.18632/oncotarget.25785

**Published:** 2018-07-24

**Authors:** Estella Zuccolo, Umberto Laforenza, Federica Ferulli, Giorgia Pellavio, Giorgia Scarpellino, Matteo Tanzi, Ilaria Turin, Pawan Faris, Angela Lucariello, Marcello Maestri, Dlzar Ali Kheder, Germano Guerra, Paolo Pedrazzoli, Daniela Montagna, Francesco Moccia

**Affiliations:** ^1^ Department of Biology and Biotechnology “Lazzaro Spallanzani”, University of Pavia, Pavia, Italy; ^2^ Department of Molecular Medicine, University of Pavia, Pavia, Italy; ^3^ Laboratory of Immunology Transplantation, Foundation IRCCS Policlinico San Matteo, Pavia, Italy; ^4^ Department of Biology, College of Science, Salahaddin University, Erbil, Kurdistan-Region of Iraq, Iraq; ^5^ Department of Medicine and Health Sciences “Vincenzo Tiberio”, University of Molise, Campobasso, Italy; ^6^ Unit of General Surgery, Foundation IRCCS Policlinico San Matteo, Pavia, Italy; ^7^ Department of Biology, University of Zakho, Zakho, Kurdistan-Region of Iraq, Iraq; ^8^ Medical Oncology, Foundation IRCCS Policlinico San Matteo, Pavia, Italy; ^9^ Department of Sciences Clinic-Surgical, Diagnostic and Pediatric, University of Pavia, Pavia, Italy

**Keywords:** colorectal cancer, store-operated Ca^2+^ entry, Stim, Orai, proliferation

## Abstract

Store-operated Ca^2+^ entry (SOCE) provides a major Ca^2+^ entry route in cancer cells. SOCE is mediated by the assembly of Stim and Orai proteins at endoplasmic reticulum (ER)-plasma membrane junctions upon depletion of the ER Ca^2+^ store. Additionally, Stim and Orai proteins underpin constitutive Ca^2+^ entry in a growing number of cancer cell types due to the partial depletion of their ER Ca^2+^ reservoir. Herein, we investigated for the first time the structure and function of SOCE in primary cultures of colorectal carcinoma (CRC) established from primary tumor (pCRC) and metastatic lesions (mCRC) of human subjects. Stim1-2 and Orai1-3 transcripts were equally expressed in pCRC and mCRC cells, although Stim1 and Orai3 proteins were up-regulated in mCRC cells. The Mn^2+^-quenching technique revealed that constitutive Ca2^+^ entry was significantly enhanced in pCRC cells and was inhibited by the pharmacological and genetic blockade of Stim1, Stim2, Orai1 and Orai3. The larger resting Ca^2+^ influx in pCRC was associated to their lower ER Ca^2+^ content as compared to mCRC cells. Pharmacological and genetic blockade of Stim1, Stim2, Orai1 and Orai3 prevented ER-dependent Ca^2+^ release, thereby suggesting that constitutive SOCE maintains ER Ca^2+^ levels. Nevertheless, pharmacological and genetic blockade of Stim1, Stim2, Orai1 and Orai3 did not affect CRC cell proliferation and migration. These data provide the first evidence that Stim and Orai proteins mediate constitutive Ca^2+^ entry and replenish ER with Ca^2+^ in primary cultures of CRC cells. However, SOCE is not a promising target to design alternative therapies for CRC.

## INTRODUCTION

Colorectal carcinoma (CRC) is the most common gastrointestinal cancer in the world [1] and, at early stage, surgical resection followed by adjuvant chemotherapy in higher risk population to eradicate distant micrometastases is the standard treatment when the disease is diagnosed [2]. In those patients showing disease recurrence or being metastatic at diagnosis, 5-year survival falls dramatically from 80-90% to 10%-20% despite development of effective combinations of chemotherapeutic agents along with the introduction of targeted therapies including monoclonal antibodies against epidermal growth factor receptor or vascular endothelial growth factor (VEGF) [2]. This makes of pivotal importance the search for alternative molecular targets with the ultimate goal of ameliorating the prognosis of these patients. Emerging evidence showed that extracellular Ca^2+^ entry plays a key role in promoting tumorigenesis and resistance to anticancer treatments [3, 4]. Particularly, store-operated Ca^2+^ entry (SOCE), which is a ubiquitous Ca^2+^ entry pathway in both excitable and non-excitable cells [5], regulates tumor growth and metastasis in a number of cancer cell lines [6–8], including several CRC cell lines [9–11]. SOCE is activated by either pharmacological or physiological Ca^2+^ depletion of the endoplasmic reticulum (ER) [5, 8], the main intracellular Ca^2+^ store. A large fall in ER Ca^2+^ levels activates Stim1, an ER Ca^2+^ sensor which then undergoes a complex molecular rearrangement culminating in its oligomerization and translocation to ER-plasma membrane junctions, known as *puncta*. Herein, Stim1 binds to and gates Orai1, the pore-forming subunit of store-operated calcium channels (SOCCs), thereby triggering SOCE [5]. In addition to Orai1, its close paralogue, Orai3, has been shown to mediate Stim1-triggered SOCE in oestrogen receptor positive (ER^+^) breast cancer [12] and non-small cell lung adenocarcinoma [13] cell lines. The ER Ca^2+^ concentration is, however, quite low in cells residing within tumor microenvironment [4, 14]. Therefore, Stim1 and Orai1 may be also activated in resting, i.e. not stimulated, tumor- and tumor-associated cells [15–19], thereby mediating a constitutive influx of Ca^2+^. It should, however, be pointed out that Orai1 is constitutively open independently on Orai1, and therefore on ER Ca^2+^ levels, in both metastatic [18] and non-metastatic [17] breast cancer cells. In addition, the lipid mediator arachidonic acid (AA) promotes the store-independent interaction of Orai1 with Orai3 in prostate cancer cell lines, which leads to SOCE down-regulation and promotes cell proliferation [20]. As a consequence, Stim1, Orai1 and Orai3 have been put forward as alternative promising targets to treat untreatable and/or therapy-resistant solid cancers [6–8]. A major, yet unresolved, issue is, however, represented by the use of immortalized cancer cell lines in the majority of studies that assessed the role of SOCE in tumorigenesis. Obviously, cancer cell lines, although easy to obtain, handle and expand, cannot recapitulate the complex biology that underpins neoplastic transformation. This hurdle is further exacerbated by the growing evidence that the molecular and genetic profile of each tumor may vary from one patient to another, which led to the concept of personalized medicine to treat cancer patients [21]. Although the involvement of SOCE in tumor cell proliferation and migration has been nowadays acknowledged [6, 7], scarce information is available regarding the role of Stim and Orai proteins in patients-derived cancer cells. For instance, a recent study revealed that SOCE does not control proliferation in primary cultures of human metastatic renal cell carcinoma (RCC) [22] and glioblastoma cells [23].

Herein, we took advantage from a novel method for efficiently establishing primary cultures from CRC tissues, which retain the immune-histochemical features of the original tumor and could be isolated from both the primary tumor and its liver metastasis [24]. We demonstrated the existence of a constitutive influx of Ca^2+^ that was significantly larger in cells derived from primary tumor (pCRC) as compared to CRC cells derived from metastases (mCRC). This resting Ca^2+^ entry was sensitive to low micromolar doses of La^3+^ and Pyr6 and inhibited by the genetic suppression of Stim1, Orai1 and Orai3 and by the pharmacological inhibition of Stim2 signalling. Although this constitutive Ca^2+^ entry was enhanced in pCRC cells, Stim1 and Orai3 proteins were up-regulated in mCRC cells. The larger Ca^2+^ entry occurring in pCRC cells was associated to their lower ER Ca^2+^ levels respect to mCRC cells and was responsible for refilling the inositol-1,4,5-trisphosphate (InsP_3_)-sensitive ER Ca^2+^ store in both cell types. Surprisingly, the genetic and pharmacological suppression of constitutive Ca^2+^ entry did not affect proliferation and migration in pCRC and mCRC cells. These data demonstrate for the first time that, although Stim and Orai proteins mediate constitutive Ca^2+^ entry in CRC cells, they do not represent a reliable target to design alternative treatments to cure CRC patients.

## RESULTS

### Patient tumor cells

Since commercial long term CRC lines might be poorly representative of the original tumors, more relevant data might be obtained from *in vitro* experiments using as target cells CRC derived from primary tumor or from metastasis obtained during surgical resection and cultured *in vitro*. Primary cultures were considered successful when they met the following criteria [24, 25]: i) presence of an almost pure line of tumor cells as confirmed by morphological and immunohistochemical analysis; ii) sufficient number of cells to perform immunological and molecular studies; iii) high cell viability after cryopreservation.

### Stim and Orai proteins are expressed in pCRC and mCRC cells

**Figure 1:**
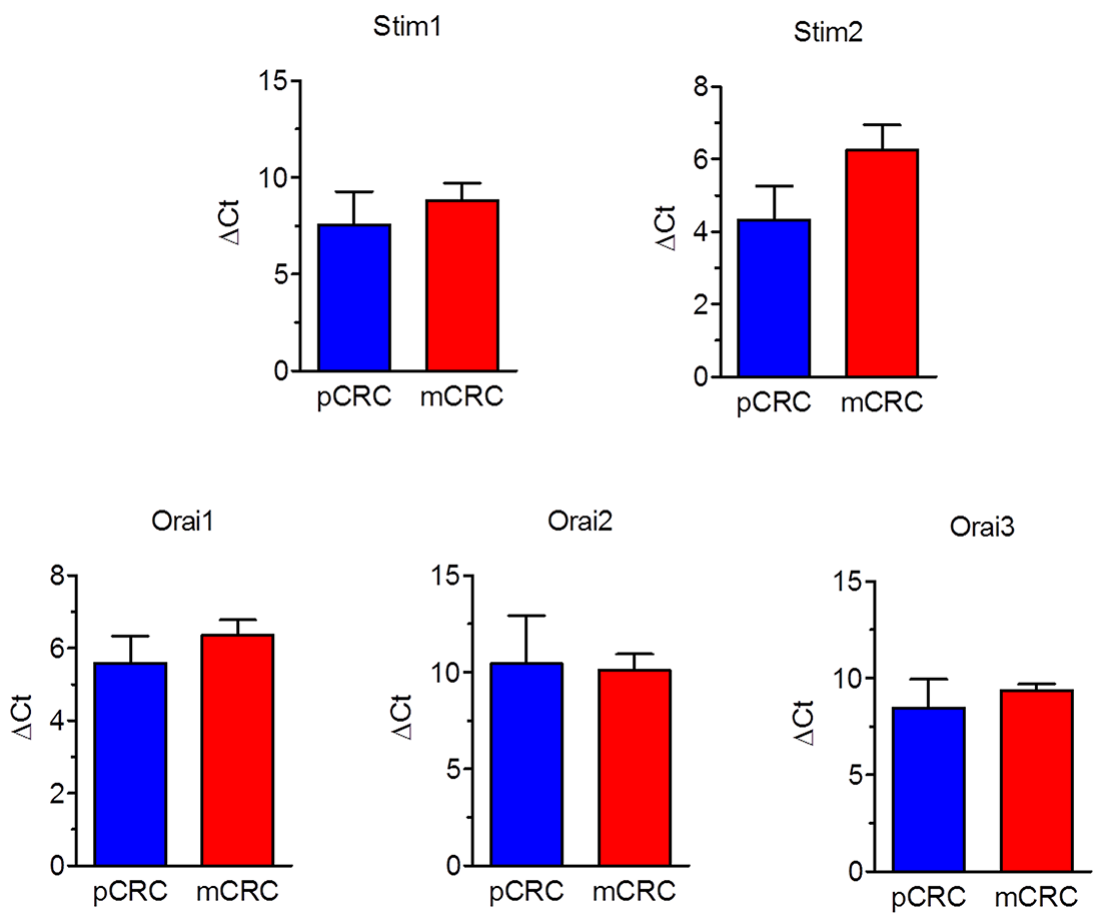
Expression of Stim1-2 and Orai1-3 transcripts in patients-derived colorectal cancer cells Quantitative real-time reverse transcription polymerase chain reaction of total RNA was performed using specific primers as indicated in Table 1. The relative mRNA levels were determined as described in Materials and Methods. In each experiment the Ct values obtained were normalized to the Ct of three housekeeping genes and then averaged. Bars represent the mean±SEM of at least 4 different RNA extracts each from different patients. The asterisk indicates p<0.05 versus pCRC (Student’s *t* test).

**Figure 2:**
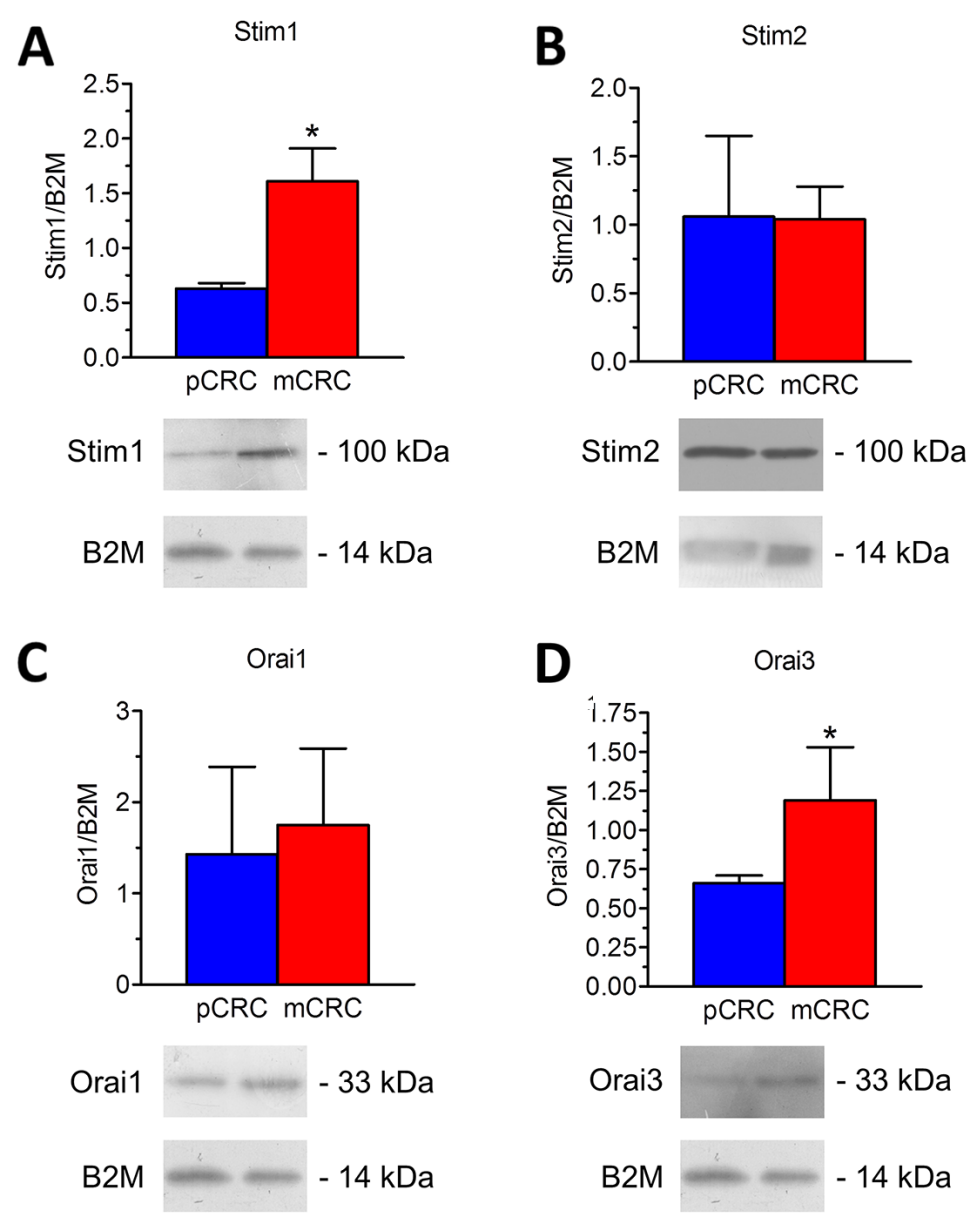
Expression of Stim1-2, Orai1 and Orai3 proteins in patients-derived colorectal cancer cells Blots representative of four (each from a distinct patient) were shown. Lanes were loaded with 30 μg of proteins, probed with affinity purified antibodies and processed as described in Materials and Methods. The same blots were stripped and re-probed with anti-beta-2-microglobulin (B2M) polyclonal antibody, as housekeeping. Major bands of the expected molecular weights were observed: Stim1 **(A)**, Stim2 **(B)**, Orai1 **(C)** and Orai3 **(D)**. Bands were acquired, densitometric analysis of the bands was performed by Total Lab V 1.11 computer program (Amersham Biosciences Europe, Italy) and the results were normalized to the corresponding B2M.

A throughout qRT-PCR and Western blot analysis was carried out to assess the expression of Stim1-2 and Orai1-3 in both pCRC and mCRC cells. All the transcripts investigated were readily detectable, as shown in Figure 1. Single bands of the expected size of cDNA fragments were amplified, whereas negative controls were performed by omitting the reverse transcriptase (not shown). The comparison of ΔCt values of the mRNAs obtained by qRT-PCR showed that there was no difference in the transcript levels of Stim1-2 and Orai1-3 between pCRC and mCRC cells (Figure 1). It is, however, been demonstrated that mRNA and protein levels of Stim and Orai genes do not always match with each other in cancer cells [9, 26]. Western blot analysis of Stim1-2, Orai1 and Orai3 expression was, therefore, carried out by employing affinity-purified antibodies, as shown in [22, 26, 27]. Orai2 was not investigated as it does not mediate Ca^2+^ entry in tumor cells [6], whereas it underlies SOCE in mouse brain neurons and endothelial cells [28] and in mouse T cells [29]. Immunoblots revealed a major band of ≈100 kDa for Stim1 (Figure 2A) and Stim2 (Figure 2B) and of ≈33 kDa for Orai1 (Figure 2C) and Orai3 (Figure 2D), as expected from their molecular sizes [5]. Unlike their corresponding transcripts, Stim1 and Orai3 were significantly (p<0.05) up-regulated in mCRC cells as compared to control cells (Figure 2A and Figure 2D). These findings indicate Stim and Orai proteins are expressed and may mediate extracellular Ca^2+^ entry in CRC cells.

In a separate set of experiments, we evaluated the expression of some members of the Transient Receptor Potential (TRP) Canonical (TRPC) subfamily, which may mediate SOCE in cancer cells [9, 30, 31]. The comparison of ΔCt values showed that TRPC3 and TRPC5 transcripts were up-regulated, while TRPC4 and TRPC5 mRNAs were down-regulated in mCRC cells (Supplementary Figure 1). Nevertheless, western blot analysis revealed that there was no difference in the expression levels of TRPC proteins between pCRC and mCRC cells. In more detail, immunoblots displayed a major band of about 92 kDa for TRPC1 (Supplementary Figure 2A), whereas TRPC3/6/7 and TRPC4 exhibited major bands of 96 kDa (Supplementary Figure 2B and Supplementary Figure 2C, respectively). Therefore, TRPC channels are expressed and have the potential to mediate extracellular Ca^2+^ entry in CRC cells.

### Constitutive SOCE is significantly larger in pCRC cells as compared to mCRC cells

**Figure 3:**
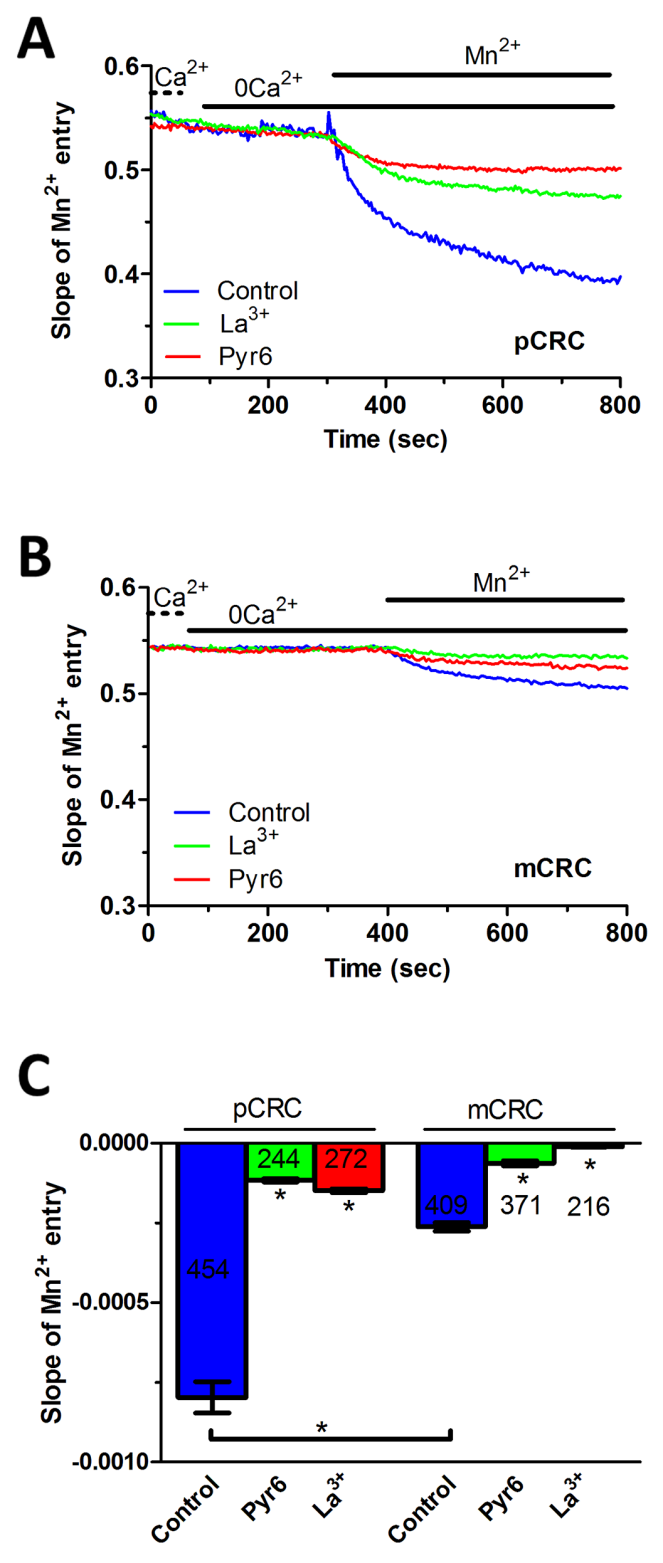
Constitutive Ca^2+^ entry in patients-derived colorectal cancer cells **(A)** resting Ca^2+^ entry in pCRC cells was evaluated by using the Mn^2+^-quenching technique as described in Materials and methods. The extracellular PSS (Ca^2+^) was first replaced with a 0Ca^2+^ solution and then 200 μM Mn^2+^ was added to cause an immediate decay in Fura-2 fluorescence, which is consistent with the occurrence of constitutive Ca^2+^ entry. Resting Mn^2+^ influx in pCRC cells was impaired by either Pyr6 (10 μM, 30 min) or La^3+^ (10 μM, 30 min). The rate of fluorescence decay for each individual tracing was calculated as the slope of a linear regression and collective data were pooled in Panel C. In these and the following figures, agonists and drugs were administered at the time indicated by the horizontal bars. **(B)** resting Ca^2+^ entry in mCRC recorded in the absence and in the presence of Pyr6 (10 μM, 30 min) or La^3+^ (10 μM, 30 min) by using the Mn^2+^-quenching technique. **(C)** mean±SE of the quenching rate of Fura-2 fluorescence induced by Mn^2+^ addition in unstimulated CRC cells under the designated treatments. The asterisk indicates p<0.05.

**Figure 4:**
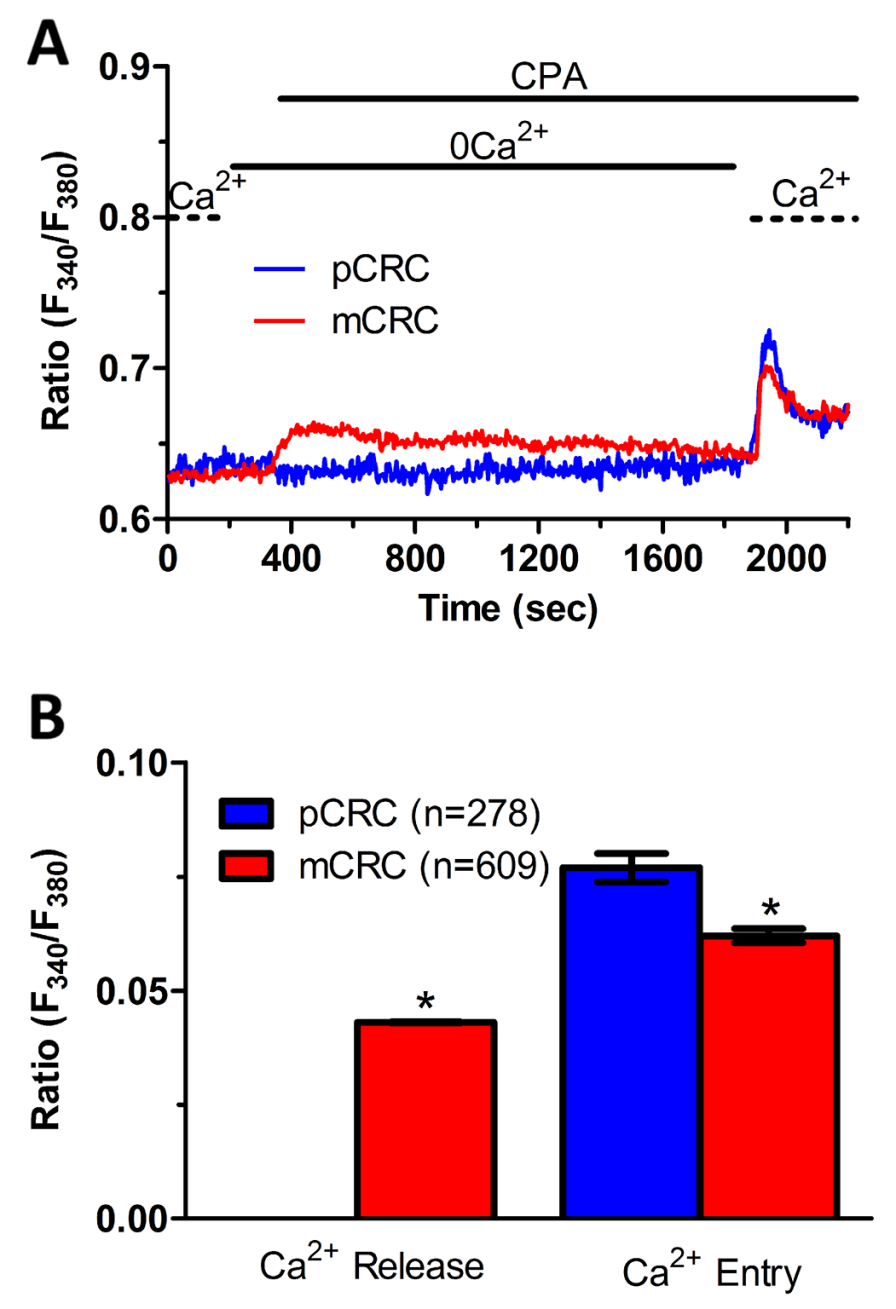
ER-dependent Ca^2+^ release is significantly reduced in primary colorectal cancer cells **(A)** the ER Ca^2+^ pool was depleted by challenging the cells with CPA (10 µM) in the absence of external Ca^2+^ (0Ca^2+^), and store-operated Ca^2+^ influx was then evaluated on Ca^2+^ replenishment to the perfusate. **(B)** mean±SE of the amplitude of CPA-induced Ca^2+^ release and CPA-induced SOCE recorded from CRC cells under the designated treatments. The asterisk indicates p<0.05.

In order to assess whether Stim and Orai proteins mediate SOCE in CRC cells, we exploited the single-cell Ca^2+^ imaging technique by loading the cells with the Ca^2+^-sensitive fluorophore, Fura-2/AM, as described for our types of cancer cells [15, 26, 27]. Our preliminary recordings showed that intracellular Ca^2+^ levels were stable in both pCRC and mCRC cells, which lacked spontaneous Ca^2+^ activity. There was no difference in resting Ca^2+^ levels between the two cell types, as the basal Fura-2/AM fluorescence was 0.84±0.009 a.u. (n=314) and 0.79±0.016 a.u. (n=150) in pCRC and mCRC cells (Supplementary Figure 3), respectively. Then, in order to assess whether they displayed a constitutive Ca^2+^ entry, we simply removed Ca^2+^ from the extracellular solution (0Ca^2+^). This maneuver caused a rapid and reversible drop in basal Ca^2+^ levels (Supplementary Figure 3), which was significantly larger in pCRC cells and was consistent with a resting Ca^2+^ permeability in both cell types. To further characterize the nature of this resting Ca^2+^ influx pathway, we turned to the Mn^2+^-quenching technique. Extracellular Mn^2+^ is able to flow through most of Ca^2+^-permeable channels, including Orai channels, thereby causing a drop in Fura-2 fluorescence, which is independent on intracellular Ca^2+^ concentration ([Ca^2+^]_i_) and is more evident at 360 nm, i.e. the isosbestic wavelength for Fura-2 [15, 17]. As shown in Figure 3, there was a clear decay in Fura-2 fluorescence upon substitution of extracellular Ca^2+^ with Mn^2+^ in both pCRC and mCRC cells, which displayed a rather linear quenching of Fura-2 fluorescence. This finding further corroborates the notion that a constitutive Ca^2+^ entry pathway is active in both cell types. As discussed elsewhere [15], the slope of the first 400 s of the quenching trace can be exploited to measure the rate of Mn^2+^ influx into the cells. Our results clearly indicate that the rate of fluorescence decay was significantly higher in pCRC as compared to mCRC cells (Figure 3). Subsequently, we evaluated the rate of fluorescence decay in the presence of either Pyr6 (10 μM, 30 min) or La^3+^ (10 μM, 30 min), two selective Orai inhibitors [5, 6]. Both drugs caused a significant (p<0.05) reduction in the slope of the constitutive Mn^2+^ influx (Figure 4). Likewise, YM-58483/BTP2 (10 μM, 30 min), which may also block Orai1 and constitutive SOCE [6, 15, 32], reduced the rate of basal Ca^2+^ entry in both pCRC and mCRC cells (Supplementary Figure 4). Therefore, resting Ca^2+^ entry is likely to be mediated by Orai channels and is enhanced in pCRC compared to mCRC cells, despite the fact that Stim1 and Orai3 were up-regulated in the latter. The rate of Mn^2+^ entry was further enhanced by depleting the ER Ca^2+^ pool with cyclopiazonic acid (CPA; 10 μM) (see also below), which indicates that resting SOCE could be boosted by extracellular stimulation (Supplementary Figure 5). Accordingly, Pyr6 (10 μM, 30 min) and La^3+^ (10 μM, 30 min) reduced CPA-induced Mn^2+^ entry in both cell types (Supplementary Figure 5) [15, 32]. Similar to constitutive SOCE, the rate of CPA-induced Mn^2+^ entry was significantly larger in pCRC cells as compared to mCRC cells (Supplementary Figure 5).

Conversely, both cell types did not show any detectable increase in [Ca^2+^]_i_ in the presence of 8 μM AA, which recruits Orai1/Orai3 heteromers in prostate cancer cells (Supplementary Figure 6) [20]. We further challenged the cells with 2-Aminoethoxydiphenyl borate (2-APB; 50 μM), which may stimulate store-operated currents mediated by heteromeric Orai1/Orai3 channels [33], but also activates Orai3 independently on Stim1 [34]. 2-APB (50 μM) caused a transient increase in [Ca^2+^]_i_ which then decayed to a sustained plateau phase in both pCRC (Supplementary Figure 7A) and mCRC cells (Supplementary Figure 7B), as recently reported in human prostate cancer cells [33]. The Ca^2+^ response to 2-APB was detectable only in the presence of extracellular Ca^2+^ (Supplementary Figure 7A and Supplementary Figure 7B), did not show any significant (p<0.05) difference in the amplitude between pCRC and mCRC cells (Supplementary Figure 7C), and was strongly reduced by 10 μM La^3+^ (Supplementary Figure 7C).

### ER Ca^2+^ content is regulated by constitutive Ca^2+^ entry and is lower in pCRC cells

**Figure 5:**
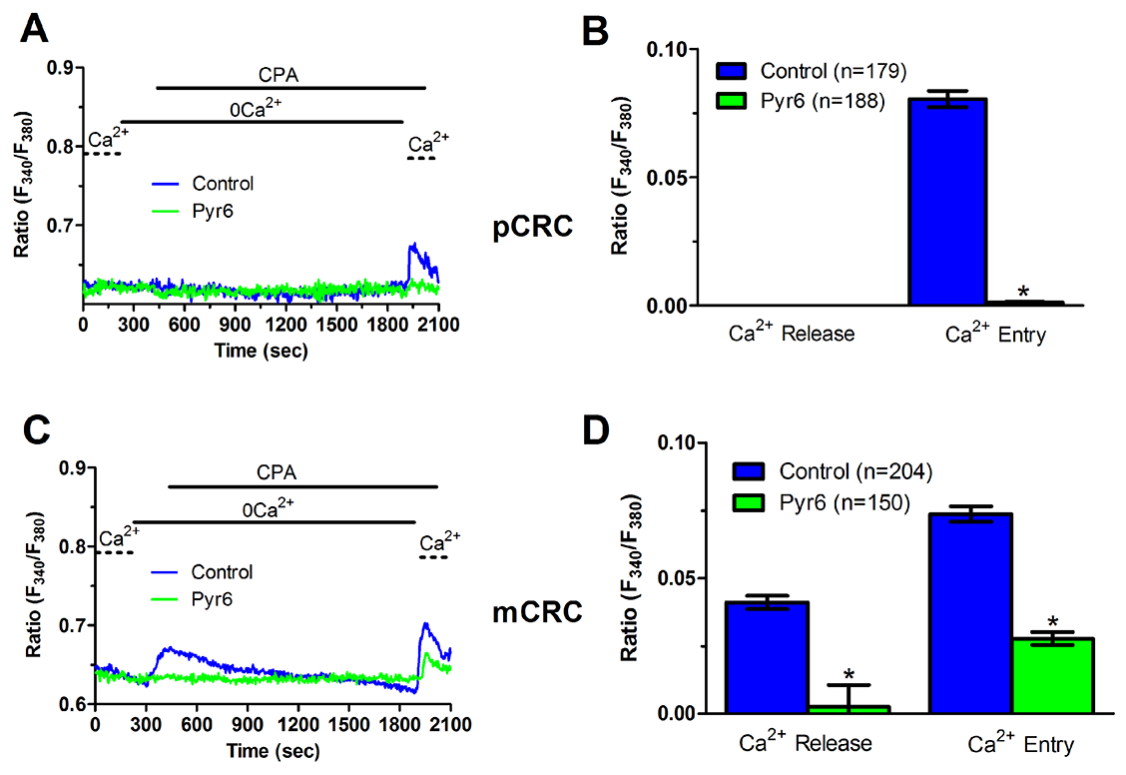
Pyr6 inhibits ER-dependent Ca^2+^ release in patients-derived colorectal cancer cells **(A)** pre-incubating pCRC cells with Pyr6 (10 μM, 30 min) prevented CPA-induced SOCE, which is likely to reflect the blockade of CPA-induced ER-dependent Ca^2+^ release (see text), in pCRC cells. CPA was administered at 10 μM. **(B)** mean±SE of the amplitude of CPA-induced Ca^2+^ release and CPA-induced SOCE in the absence and presence of Pyr6 in pCRC cells. The asterisk indicates p<0.05. **(C)** pre-incubating mCRC cells with Pyr6 (10 μM, 30 min) prevented both CPA-induced ER-dependent Ca^2+^ release and SOCE in mCRC cells. CPA was administered at 10 μM. **(D)** mean±SE of the amplitude of CPA-induced Ca^2+^ release and CPA-induced SOCE in the absence and presence of Pyr6 in mCRC cells. The asterisk indicates p<0.05.

**Figure 6:**
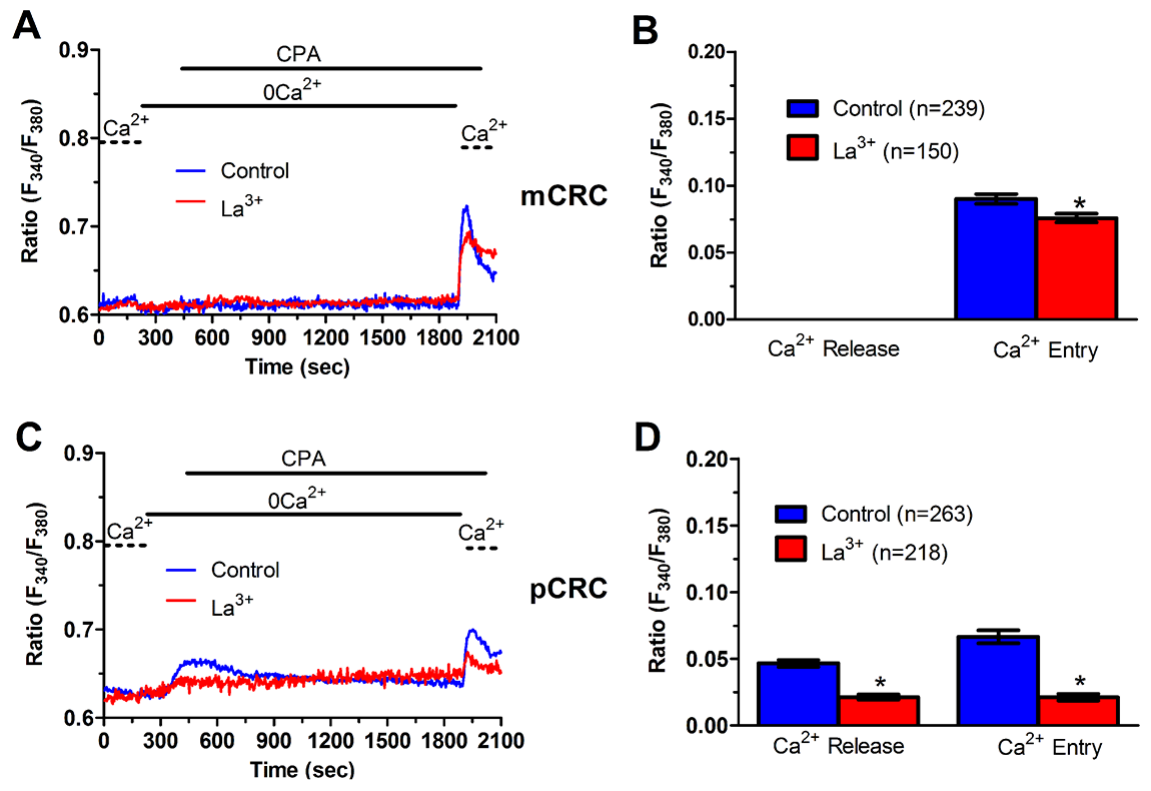
La^3+^ inhibits ER-dependent Ca^2+^ release in patients-derived colorectal cancer cells **(A)** pre-incubating pCRC cells with La^3+^ (10 μM, 30 min) reduced CPA-induced SOCE, which is likely to reflect the blockade of CPA-induced ER-dependent Ca^2+^ release (see text), in pCRC cells. CPA was administered at 10 μM. **(B)** mean±SE of the amplitude of CPA-induced Ca^2+^ release and CPA-induced SOCE in the absence and presence of La^3+^ in pCRC cells. The asterisk indicates p<0.05. **(C)** pre-incubating mCRC cells with La^3+^ (10 μM, 30 min) dramatically attenuated both CPA-induced ER-dependent Ca^2+^ release and SOCE in mCRC cells. CPA was administered at 10 μM. **(D)** mean±SE of the amplitude of CPA-induced Ca^2+^ release and CPA-induced SOCE in the absence and presence of Pyr6 in mCRC cells. The asterisk indicates p<0.05.

We have recently demonstrated that SOCE is basally activated in infantile hemangioma (IH)-derived endothelial colony forming cells (IH-ECFCs), but not normal ECFCs (N-ECFCs), despite the fact that there is no difference in the expression levels of Stim and Orai proteins. The constitutive activation of the SOCE machinery is maintained by the chronic underfilling of the ER Ca^2+^ store in IH-ECFCs [15]. Constitutive SOCE, in turn, ensures the refilling of the ER Ca^2+^ pool, as also observed in several cell types [28, 32, 35–37]. In order to assess the ER Ca^2+^ releasing ability of pCRC and mCRC cells, we exploited the “Ca^2+^ add-back” protocol [15, 27, 30, 38], which consists in challenging the cells with cyclopiazonic acid (CPA) in the absence of extracellular Ca^2+^ (0Ca^2+^). CPA is a selective inhibitor of Sarco-Endoplasmic Reticulum Ca^2+^-ATPase (SERCA) that prevents Ca^2+^ sequestration into ER lumen, thereby causing a transient increase in [Ca^2+^]_i_ due to passive ER Ca^2+^ leakage. The Ca^2+^ response to CPA in the absence of extracellular Ca^2+^ (0Ca^2+^) is a widely established tool to monitor changes in ER Ca^2+^ levels [15, 30, 35, 39]. Subsequently, extracellular Ca^2+^ is restored to the perfusate, thereby causing a second increase in [Ca^2+^]_i_ which reflects SOCE activation [15, 27, 30, 38]. We first observed that CPA failed to elicit any detectable increase in [Ca^2+^]_i_ in pCRC cells (Figure 3A), while it elicited a robust intracellular Ca^2+^ release in mCRC cells (Figure 3A). Nevertheless, the subsequent addition of extracellular Ca^2+^ to ER depleted cells elicited a second increase in [Ca^2+^]_i_, which is typical of SOCE activation [15, 22], in both pCRC and mCRC cells (Figure 3A). The statistical analysis of these results is presented in Figure 3B. It turns out that CPA was notwithstanding able to release ER Ca^2+^ also in pCRC cells, although this signal was extremely localized and not detectable by our epifluorescence system. Therefore, SOCE activation could be used as a surrogate to monitor ER Ca^2+^ mobilization in pCRC cells. Of note, CPA-evoked SOCE was still larger in pCRC cells, thereby confirming Mn^2+^ entry data. Then, we found that CPA-induced intracellular Ca^2+^ release was significantly (p<0.05) reduced in the presence of either Pyr6 (10 μM, 30 min) (Figure 5) or La^3+^ (10 μM, 30 min) (Figure 6) in pCRC, as monitored by the lack of SOCE activation, and in mCRC cells. These results strongly suggest that the lower ER Ca^2+^ concentration is likely to drive the larger SOCE recorded in resting, i.e. not stimulated pCRC cells. Furthermore, they support the widespread model according to which constitutive SOCE in CRC cells is required to replenish the ER Ca^2+^ store [15, 28, 32, 35–37].

**Figure 7:**
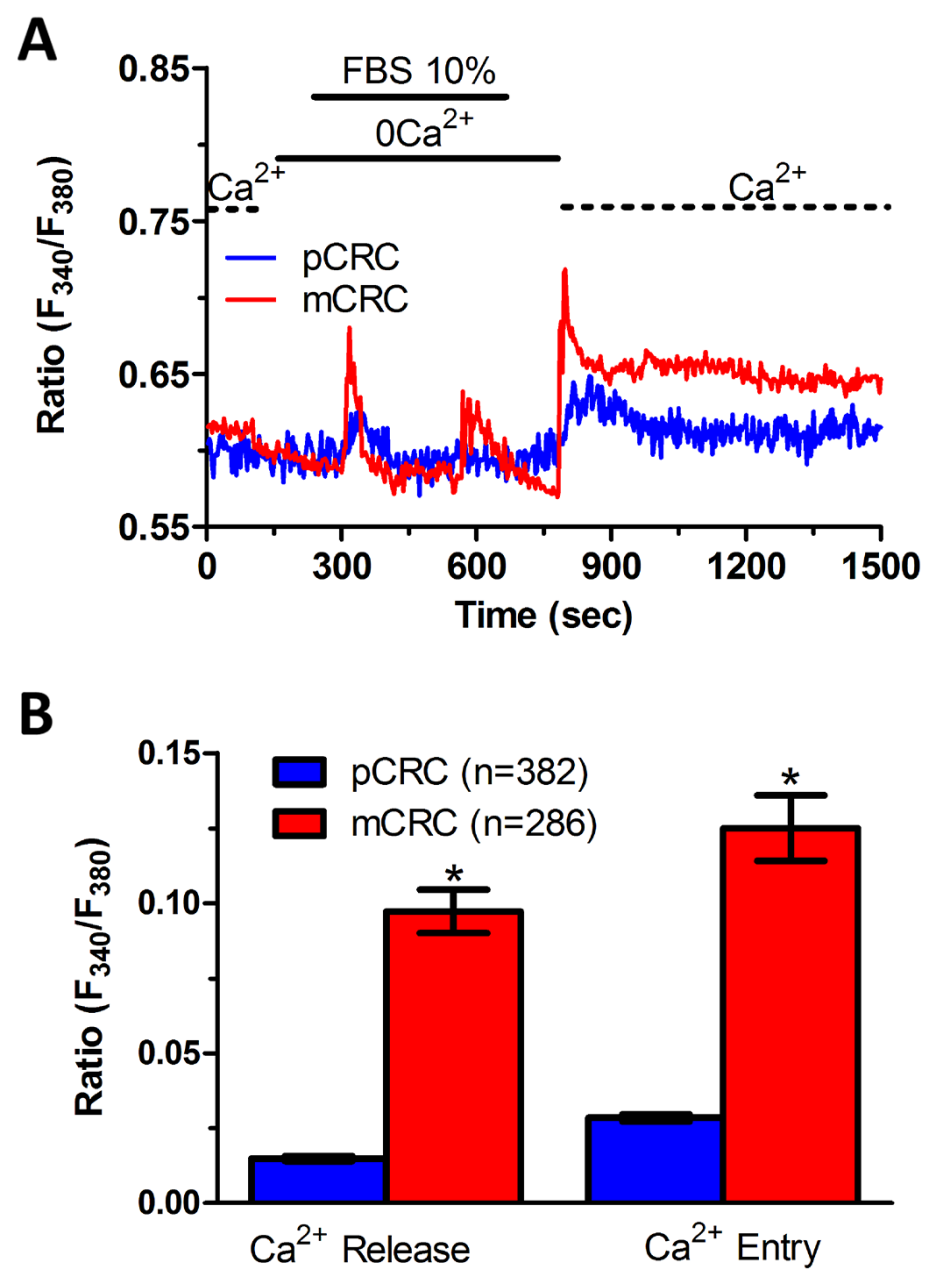
InsP_3_-dependent Ca^2+^ release is lower in primary colorectal cancer cells **(A)** 10% FBS-induced InsP_3_-dependent intracellular Ca^2+^ release and SOCE were remarkably lower in pCRC cells as compared to mCRC cells. **(B)** mean±SE of the amplitude of 10% FBS-induced intracellular Ca^2+^ release and SOCE in pCRC and mCRC cells. The asterisk indicates p<0.05.

**Figure 8:**
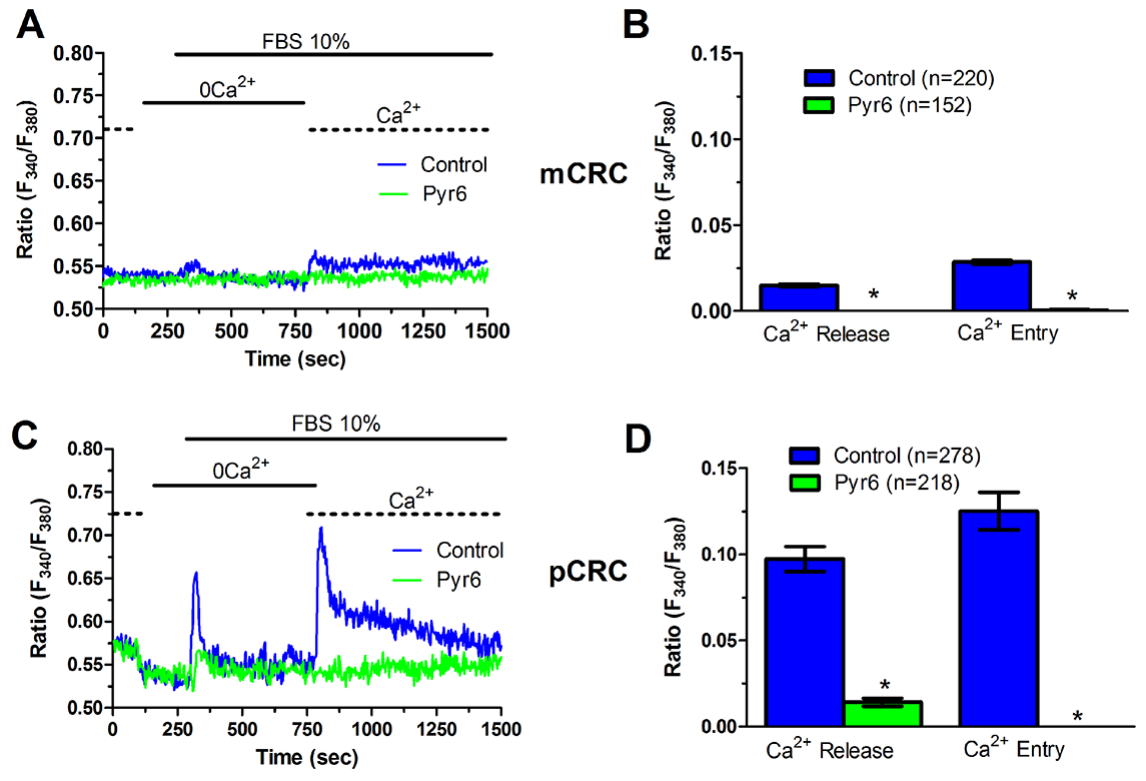
Pyr6 inhibits InsP_3_-dependent Ca^2+^ release in patients-derived colorectal cancer cells **(A)** pre-incubating pCRC cells with Pyr6 (10 μM, 30 min) prevented 10% FBS-induced InsP_3_-dependent Ca^2+^ release and SOCE. **(B)** mean±SE of the amplitude of 10% FBS-induced intracellular Ca^2+^ release and SOCE in the absence and presence of Pyr6 in pCRC cells. The asterisk indicates p<0.05. **(C)** pre-incubating mCRC cells with Pyr6 (10 μM, 30 min) prevented 10% FBS-induced intracellular Ca^2+^ release and SOCE in mCRC cells. **(D)** mean±SE of the amplitude of 10% FBS-induced intracellular Ca^2+^ release and SOCE in the absence and presence of Pyr6 in mCRC cells. The asterisk indicates p<0.05.

**Figure 9:**
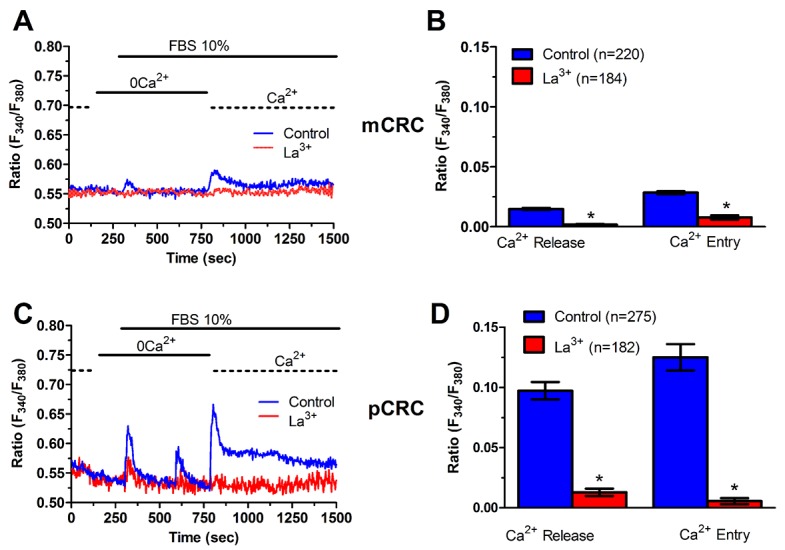
La^3+^ inhibits InsP_3_-dependent Ca^2+^ release in patients-derived colorectal cancer cells **(A)** pre-incubating pCRC cells with La^3+^ (10 μM, 30 min) prevented 10% FBS-induced InsP_3_-dependent Ca^2+^ release and SOCE in pCRC cells. **(B)** mean±SE of the amplitude of 10% FBS-induced InsP_3_-dependent Ca^2+^ release and SOCE in the absence and presence of La^3+^ in pCRC cells. The asterisk indicates p<0.05. **(C)** pre-incubating mCRC cells with La^3+^ (10 μM, 30 min) prevented 10% FBS-induced InsP_3_-dependent Ca^2+^ release and SOCE. **(D)** mean±SE of the amplitude of 10% FBS-induced InsP_3_-dependent intracellular in the absence and presence of La^3+^ in mCRC cells. The asterisk indicates p<0.05.

In order to more deeply investigate, we challenged CRC cells with adenosine 5′-trisphosphate (ATP) to assess whether constitutive SOCE also controls the InsP_3_-sensitive ER Ca^2+^ pool. ATP may accumulate within tumor microenvironment [40] and it has long been known to cause intracellular Ca^2+^ release by activating metabotropic P_2Y_ receptors to synthesize InsP_3_ [15, 22]. Unfortunately, neither pCRC nor mCRC cells generated a reproducible Ca^2+^ signal in response to extracellular ATP (100 μM) (Supplementary Figure 8). Therefore, we turned to FBS, which is supplemented to the culture medium to promote CRC proliferation and is known to promote intracellular Ca^2+^ signaling in a myriad of cell types [22, 41]. Accordingly, FBS is a rich source of growth factors that bind to tyrosine kinase receptors to stimulate phospholipase C- (PLC) to synthesize InsP_3_ from the membrane phospholipid, phosphatidylinositol 4,5-bisphosphate (PIP_2_) [41]. 10% FBS induced substantial Ca^2+^ release in both pCRC and mCRC cells, whereas the amplitude of the Ca^2+^ peak was significantly (p<0.05) larger in the latter (Figure 7), as expected by their greater ER Ca^2+^ pool. The intracellular Ca^2+^ response to FBS was due to InsP_3_-dependent Ca^2+^ mobilization as it was abolished by 1) U73122 (10 μM, 20 min) (Supplementary Figure 9A and Supplementary Figure 10A), a selective PLC antagonist, but not by its inactive analogue U73343 (Supplementary Figure 9B and Supplementary Figure 10B); 2) 2-APB (50 μM, 30 min), which selectively blocks InsP_3_Rs under 0Ca^2+^ conditions [6, 15] (Supplementary Figure 9C and Supplementary Figure 10C); and 3) depletion of the ER Ca^2+^ pool with CPA (10 μM, 30 min) (Supplementary Figure 9D and Supplementary Figure 10D). Immunoblots revealed that there was no difference in InsP_3_R protein expression between pCRC and mCRC cells (Supplementary Figure 11), thereby confirming that the larger InsP_3_-dependent Ca^2+^ release in the latter was due to their greater ER Ca^2+^ content. Similar to CPA, pharmacological blockade of constitutive Ca^2+^ entry with either Pyr6 (10 μM, 30 min) or La^3+^ (10 μM, 30 min) inhibited both 10% FBS-induced Ca^2+^ release and 10% FBS-induced SOCE (Figure 8 and Figure 9). Overall, these findings showed that physiological InsP_3_-dependent Ca^2+^ release was enhanced in mCRC cells and suggested that it was regulated by constitutive SOCE in both cell types. Surprisingly, 10% FBS-induced SOCE was significantly larger in mCRC as compared to pCRC cells (Figure 7), whereas it was inhibited by either Pyr6 (10 μM, 30 min; Figure 8) or La^3+^ (10 μM, 30 min; Figure 9). It should, however, be pointed out that Pyr6 and La^3+^ prevent ER Ca^2+^ refilling, thereby inhibiting ER-dependent Ca^2+^ release, which in turn impairs full agonists (i.e. CPA and FBS)-induced SOCE activation. It is not clear why Ca^2+^ entry is enhanced when SOCE is triggered by physiological (i.e. InsP_3_) rather than pharmacological (i.e. CPA) stimuli, but InsP_3_Rs could be more tightly coupled to the SOCE machinery than ER leakage channels [42] or InsP_3_-dependent Ca^2+^ release could cause a larger ER Ca^2+^ depletion than CPA [9].

### Stim1, Stim2, Orai1 and Orai3 mediate constitutive Ca^2+^ entry in mCRC cells

**Figure 10:**
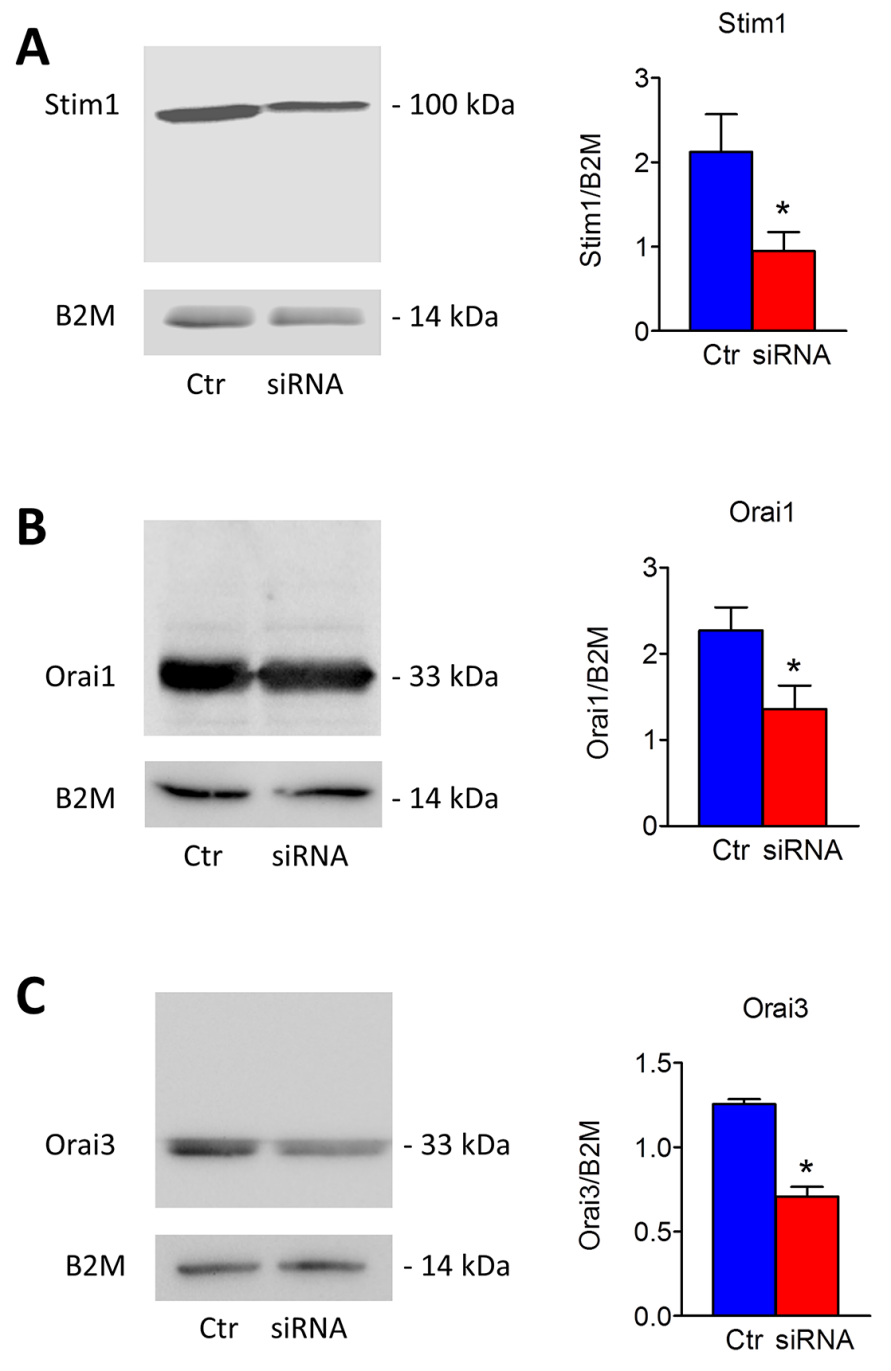
Silencing of Stim1, Orai1 and Orai3 in mCRC cells Short interfering RNA (siRNA) and scrambled siRNA (Ctr) were transfected in mCRC cells as described in Materials and methods. Western blot and densitometry demonstrate a significant reduced Stim1 **(A)**, Orai1 **(B)** and Orai3 **(C)** protein expression in silenced cells compared to controls (^*^, p<0.05; Student’s *t* test). Blots representative of three were shown. Lanes were loaded with 30 μg of proteins, probed with affinity purified antibodies and processed as described in Materials and Methods. The same blots were stripped and re-probed with anti-beta-2-microglobulin (B2M) antibody. Bands of the expected molecular weights were shown. Bands were acquired with the Image Master VDS (Amersham Biosciences Europe, Italy). Densitometric analysis of the bands was performed by Total Lab V 1.11 computer program (Amersham Biosciences Europe, Italy) and the results were normalized to the corresponding B2M.

**Figure 11:**
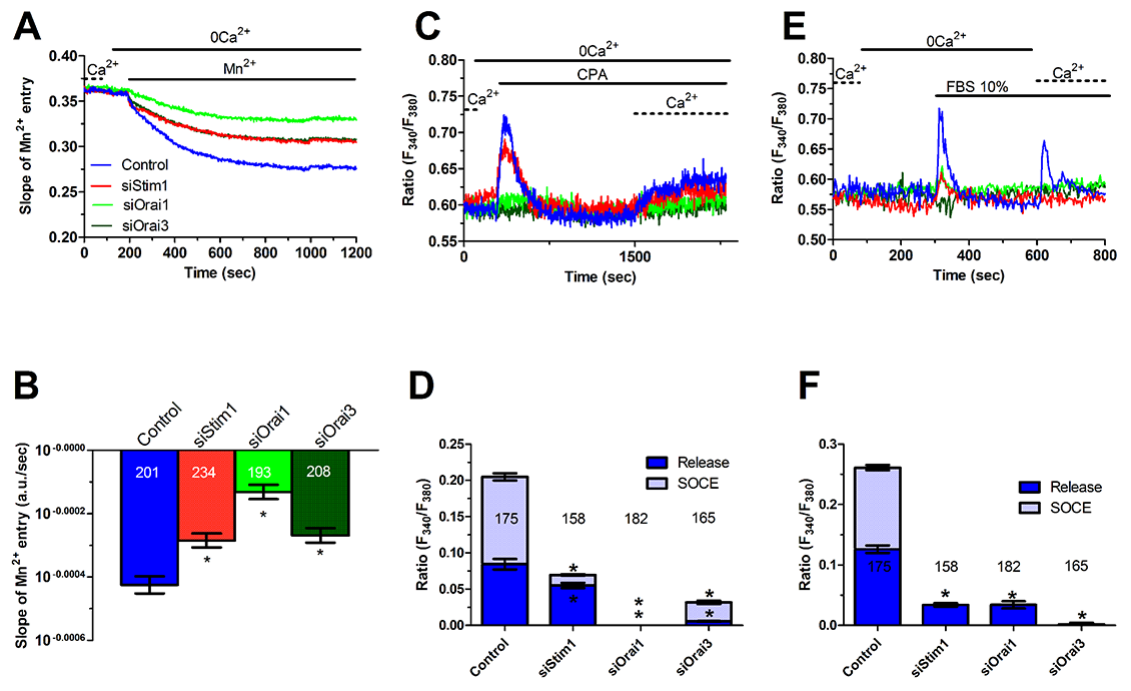
Genetic silencing of Stim1, Orai1 and Orai3 reduces constitutive Ca^2+^ entry and reduces ER Ca^2+^ refilling in metastatic colorectal cancer cells **(A)** resting Mn^2+^ influx in mCRC cells was attenuated by the genetic deletion of Stim1, Orai1 or Orai3 proteins, as described in Figure 10. **(B)** mean±SE of the quenching rate of Fura-2 fluorescence induced by Mn^2+^ addition in control mCRC cells and in mCRC cells silenced for Stim1, Orai1 or Orai1 proteins. The asterisk indicates p<0.05. **(C)** genetic silencing of Stim1, Orai1 or Orai3 proteins reduced (i.e. Stim1) or remarkably attenuated (i.e. Orai1 and Orai3) 10 μM CPA-induced ER-dependent Ca^2+^ release in mCRC cells. **(D)** mean±SE of the amplitude of CPA-induced Ca^2+^ release in mCRC cells under the designated treatments. The asterisk indicates p<0.05. **(E)** genetic silencing of Stim1, Orai1 or Orai3 proteins reduced 10% FBS-induced InsP_3_-dependent intracellular Ca^2+^ release in mCRC cells. **(F)** mean±SE of the amplitude of 10% FBS-induced Ins_P3_ intracellular Ca^2+^ release in mCRC cells under the designated treatments. The asterisk indicates p<0.05.

**Figure 12:**
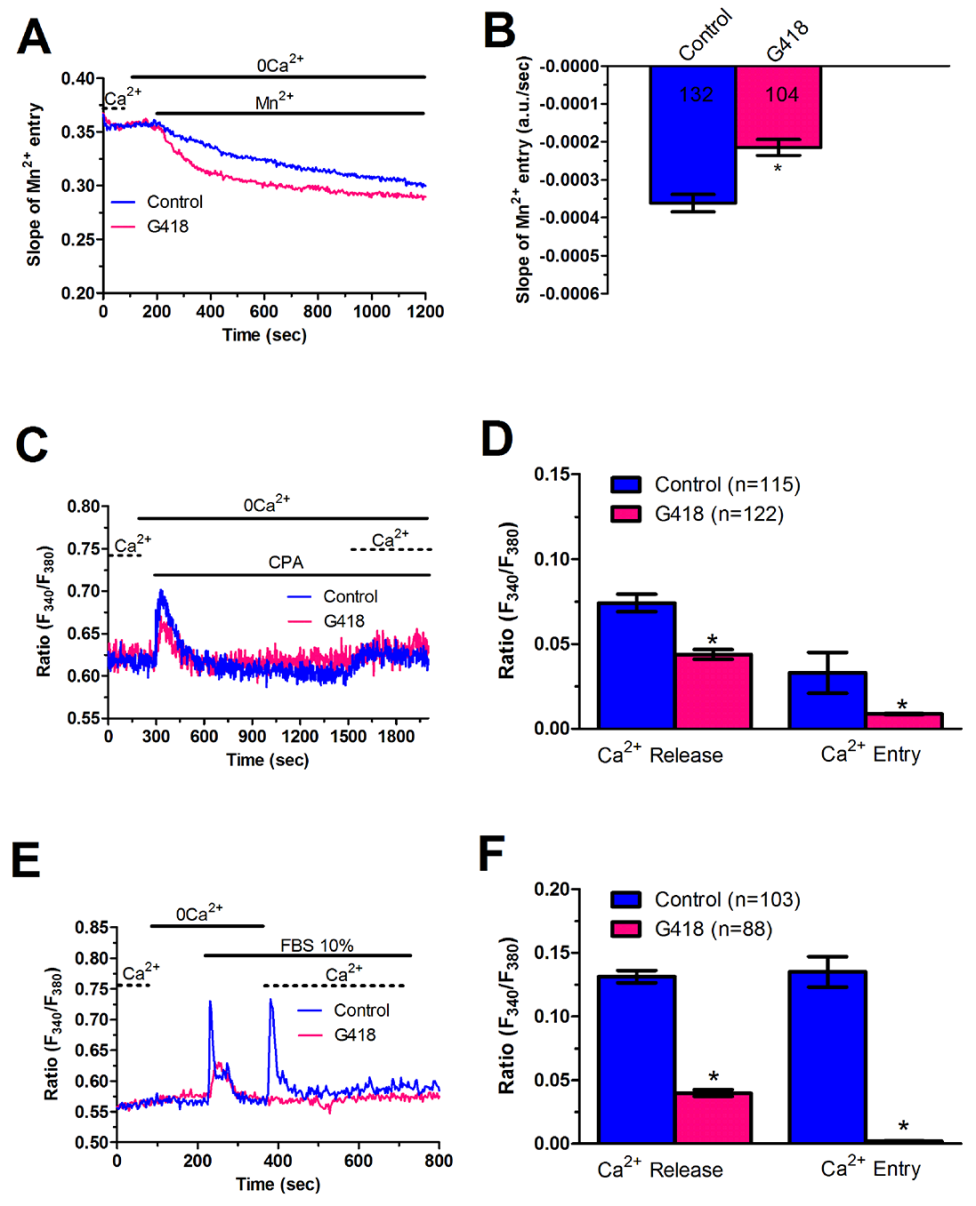
G418 inhibits constitutive Ca^2+^ entry and reduces InsP_3_-dependent Ca^2+^ release in metastatic colorectal cancer cells **(A)** resting Mn^2+^ influx in mCRC cells incubated in the absence and presence of G418 (500 μg/ml; 2 days), an established inhibitor of Stim2-dependent SOCE. **(B, C)** mean±SE of the quenching rate recorded in mCRC cells pre-incubated in the absence or in the presence of G418 (500 μg/ml; 2 days). The asterisk indicates p<0.05. (C) G418 (500 μg/ml; 2 days) reduced CPA-induced ER-dependent Ca^2+^ release in mCRC cells. **(D)** mean±SE of the amplitude of CPA-induced Ca^2+^ release in mCRC cells in the absence and in the presence of G418. The asterisk indicates p<0.05. **(E)** G418 (500 μg/ml; 2 days) attenuated 10% FBS-induced InsP_3_-dependent Ca^2+^ release in mCRC cells. **(F)** mean±SE of the amplitude of 10% FBS-induced InsP_3_ Ca^2+^ release in mCRC cells in the absence and in the presence of G418. The asterisk indicates p<0.05.

In order to unravel the molecular underpinnings of constitutive Ca^2+^ entry, we undertook protein knockdown of Stim1, Orai1 and Orai3, that have been involved in SOCE activation in cancer cells (Moccia et al., 2015; Vashisht et al., 2015), by using a siRNA silencing approach. We focussed on mCRC cells for the following reasons: 1) metastasis is the leading reason for mortality in cancer patients [43] and 2) Stim1 and Orai3 proteins were up-regulated in mCRC cells (Figure 2). The efficacy of Stim1, Orai1 and Orai3 downregulation was validated at protein level. Western blot analysis conducted with anti-Stim1, anti-Orai1 and anti-Orai3 antibodies at 48 h post-transfection revealed that Stim1 (Figure 10A), Orai1 (Figure 10B), and Orai3 (Figure 10C) expression decreased by ≈50% for each protein. Fura-2/AM imaging demonstrated that resting Mn^2+^ entry was significantly (p<0.05) reduced by knockdown of Stim1, Orai1 or Orai3 in mCRC cells (Figure 11A and Figure 11B). Furthermore, each siRNA reduced both CPA- (Figure 11C and Figure 11D) and InsP_3_-induced (Figure 11E and Figure 11F) intracellular Ca^2+^ release and SOCE. The contribution of Stim2 to constitutive Ca^2+^ entry was evaluated by pre-treating mCRC cells with G418, an aminoglycoside antibiotic that blocks Stim1 signalling without interfering with Stim1 [15, 44]. G418 (100 μM, 48 h) reduced resting Mn^2+^ entry (Figure 12A and Figure 12B), CPA- (Figure 12C-Figure 12D) and InsP_3_-induced (Figure 12E-Figure 12F) intracellular Ca^2+^ release and SOCE. Overall, these data demonstrate that Stim1, Stim2, Orai1 and Orai3 mediate resting Ca^2+^ entry in CRC cells, which may therefore be attributed to constitutive SOCE activation. Moreover, Stim1, Stim2, Orai1 and Orai3 regulate ER Ca^2+^ content in CRC cells and regulate the magnitude of physiologically induced InsP_3_-sensitive Ca^2+^ release. As observed earlier for Pyr6 and La^3+^, the genetic inhibition of constitutive SOCE with specific siRNAs (Stim1, Orai1 and Orai3) and with G418 (Stim2) is likely to reduce agonists (i.e. CPA and FBS)-induced SOCE by preventing ER Ca^2+^ refilling. Finally, we showed that genetic deletion of Stim1, Orai1 and Orai3 significantly (p<0.05) reduced 2-APB-evoked Ca^2+^ entry in mCRC cells (Supplementary Figure 7D and Supplementary Figure 7E).

### Stim1, Stim2, Orai1 and Orai3 do not mediate proliferation and migration in mCRC cells

**Figure 13:**
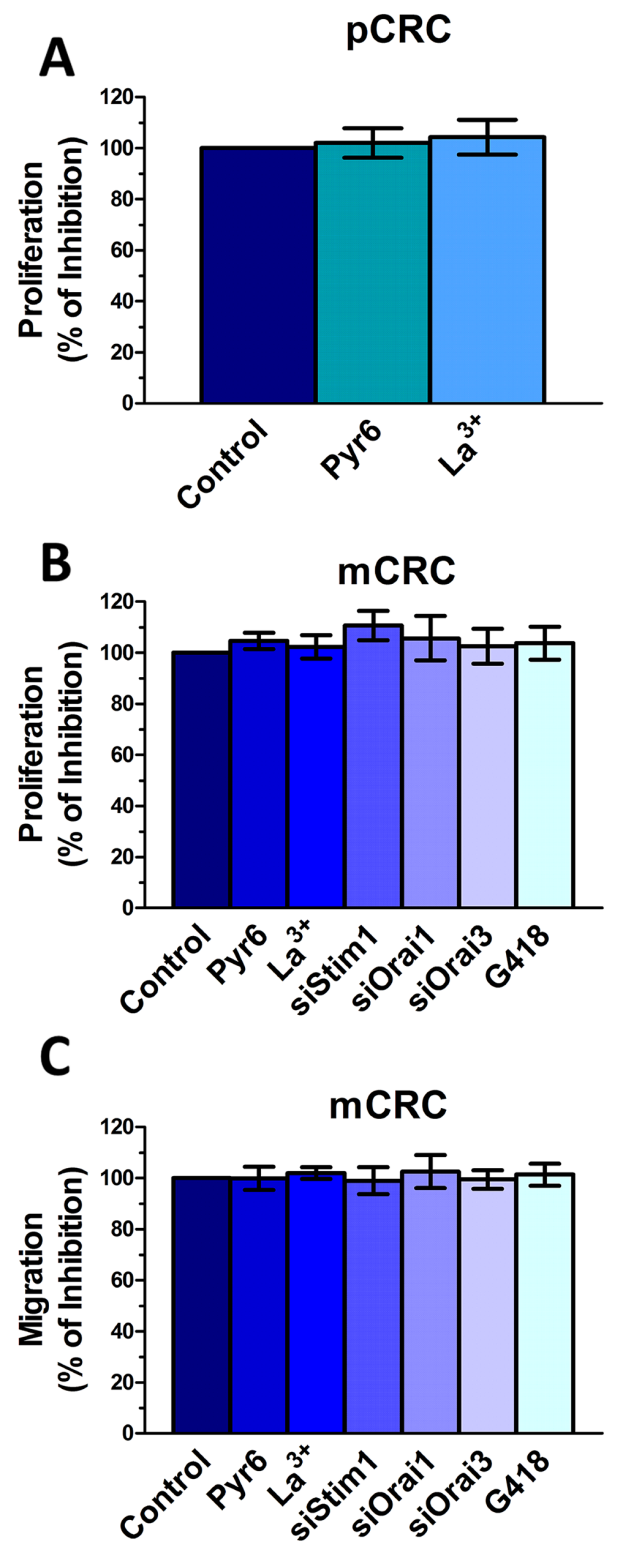
Genetic and pharmacological inhibition of constitutive Ca^2+^ entry does not affect proliferation and migration in colorectal cancer cells **(A)** preincubating the cells with Pyr6 (10 μM) or La^3+^ (10 μM) did not affect pCRC cell proliferation. **(B)** mCRC cell proliferation was not affected by pharmacological (10 μM Pyr6, 10 μM La^3+^ or 500 μg/ml G418) or genetic (siStim1, siOrai1 or siOrai3) inhibition of the molecular components of constitutive Ca^2+^ entry. **(C)** mCRC cell migration was not affected by pharmacological (10 μM Pyr6, 10 μM La^3+^ or 500 μg/ml G418) or genetic (siStim1, siOrai1 or siOrai3) inhibition of the molecular components of constitutive Ca^2+^ entry. The procedures for the proliferation and migration assays have been described in Materials and methods.

Stim and Orai proteins were shown to regulate proliferation and migration in a multitude of cancer cell lines [6, 7]. Quite surprisingly, however, the genetic inhibition of Stim1, Orai1 and Orai3, and the pharmacological inhibition of Stim2 signalling with G418 did not affect mCRC cell proliferation and migration (Figure 13). Consistently, neither Pyr6 (10 μM, 30 min) nor La^3+^ (10 μM, 30 min) affected proliferation and migration in CRC cells (Figure 13). Therefore, Stim and Orai proteins do not drive proliferation and migration in patients-derived CRC cells. These results argue against the role of Stim and Orai in CRC tumorigenesis, which has been suggested by a number of studies conducted on immortalized CRC cell lines [6, 45, 46]. Therefore, we evaluated the effect of Pyr6 (10 μM, 30 min) and La^3+^ (10 μM, 30 min) on SW480 cells, which represent a widely employed cellular model of highly aggressive CRC. Unlike patients-derived cells, Pyr6 and La^3+^ significantly (p<0.05) inhibited proliferation and migration in SW480 cells (Supplementary Figure 12). These data strongly suggest that the function of SOCE in CRC cells could be overrated if only studied in immortalized cell lines, at least for what concerns proliferation and migration.

## DISCUSSION

The present study investigated for the first time the expression, molecular structure and role of SOCE in patients-derived CRC cells. While the remodeling of Ca^2+^ signalling in cancer has been firmly established [4, 47], the Ca^2+^ toolkit is far from being exploited as an effective target for anticancer treatments due to the scarce availability of patients-derived cells, which are obviously more complicated to harvest and expand as compared to cancer cell lines [6, 48]. Preliminary reports, however, revealed that SOCE does not promote proliferation in primary cultures of patients-derived cells [22, 23]. Herein, we provided the evidence that Stim1-2, Orai1 and Orai3 proteins mediate constitutive Ca^2+^ entry in both primary and metastatic CRC cells deriving from human tumor samples. Although Stim1 and Orai3 proteins are remarkably upregulated in mCRC cells, the magnitude of constitutive Ca^2+^ entry is significantly larger in pCRC, most likely due to the partial depletion of their ER Ca^2+^ stores. Constitutive SOCE controls ER Ca^2+^ refilling, but do not drive CRC cell proliferation and migration, thus suggesting that Stim and Orai proteins are not a promising target to treat CRC.

Earlier studies demonstrated that Stim and Orai proteins are widely expressed in human CRC samples. Stim1 protein was upregulated in CRC tissues as compared to adjacent non-tumor tissues [46], whereas Stim2 transcripts were over-expressed in a well annotated cohort of CRC patients treated with adjuvant 5-FU chemotherapy [49]. Likewise, Orai1 protein was largely expressed in primary CRC samples, but not in adjacent non-tumor tissues [50]. Herein, we found that Stim1-2 and Orai1-3 transcripts were equally expressed in pCRC and mCRC cells, although Stim1 and Orai3 proteins were significantly up-regulated in mCRC cells. This result is similar to that reported by Sobradillo and coworkers, who found that Orai1 and Orai3 proteins were over-expressed in the HT29 CRC cell line, although they showed no change at transcript level [9]. Likewise, Stim1 and Orai1 proteins in primary myelofibrosis-derived ECFCs were significantly increased relative to N-ECFCs, although there was no difference in their transcript levels [26]. Moreover, the larger magnitude of agonist-induced SOCE in the human glioblastoma cell line U251 was not accompanied by the up-regulation of the mRNAs encoding for Stim1 and Orai1 [23]. It has earlier been proposed that the protein expression of the manifold components of the Ca^2+^ toolkit could be finely regulated by controlling mRNA stability [51]. For instance, the slower rate of SERCA2A mRNA degradation causes a 100-fold increase in SERCA2A protein expression in left ventricular myocytes as compared to stomach smooth muscle [52]. An alternative, but not mutually exclusive mechanism, involves an increase in the rate of protein synthesis in mCRC relative to pCRC cells, which could lead to the selective up-regulation of Stim1 and Orai3 in the former [53].

Stim and Orai proteins mediate constitutive Ca^2+^ entry in several tumor and tumor-associated cells [15–19, 54]. Constitutive Ca^2+^ entry in primary cultures of pCRC and mCRC was unveiled by the reversible fall in Fura-2 fluorescence caused by removing extracellular Ca^2+^. The same protocol unmasked the existence of resting Ca^2+^ entry also in non-metastatic [17] and metastatic breast cancer cell lines, MDA-MB-435 and MDA-MB-468 [18, 19]. The fall in cytosolic Ca^2+^ levels was significantly larger in pCRC cells, but this approach does not carefully evaluate the basal Ca^2+^ permeability as incoming Ca^2+^ may be rapidly sequestered into ER lumen by SERCA activity [55–57]. Conversely, the Mn^2+^-quenching provides a much more reliable strategy to monitor resting Ca^2+^ entry as Mn^2+^ flows through most of Ca^2+^-permeable channels, including Orai, but is not captured by SERCA [15, 17, 32]. Mn^2+^-induced quenching of intracellular preloaded Fura-2 confirmed that resting Ca^2+^ entry was significantly higher in pCRC as compared to mCRC cells. Although CRC cells may also express multiple TRPC channels, some of which could mediate SOCE in cancer cells [9, 30, 31], the following pieces of evidence demonstrate that Stim and Orai proteins mediate constitutive Ca^2+^ entry and replenish the ER Ca^2+^ reservoir in primary cultures of pCRC and mCRC cells. First, resting Ca^2+^ entry was inhibited by Pyr6 and 10 μM La^3+^, two selective Orai inhibitors, which do not affect TRPC channels [5, 6, 12, 23]. Second, the genetic silencing of Stim1, Orai1 and Orai3 and the pharmacological blockade of Stim2 signalling with G418 mimicked the inhibitory effect of Pyr6 and 10 μM La^3+^. Therefore, Stim1, Stim2, Orai1 and Orai3 mediate constitutive Ca^2+^ entry in CRC cells. Third, the ER Ca^2+^ store, as evaluated by the amplitude of the intracellular Ca^2+^ response to CPA and the InsP_3_-synthesizing agonist FBS, was significantly down-regulated in pCRC cells as compared to mCRC cells. This observation strongly correlates the rate of basal Ca^2+^ entry with the degree of store depletion and helps to understand why constitutive SOCE is significantly larger in pCRC cells, which show a partially depleted ER, despite the fact that Stim1 and Orai3 proteins are upregulated in mCRC cells. A similar mechanism has been described in CRC cell lines, in which the threshold of SOCE activation is at rapid reach upon ER Ca^2+^ depletion, while normal colon mucosa cell line display only modest SOCE in response to extracellular stimulation due to their greater ER Ca^2+^ content [9]. It could, therefore, be envisaged that the main function of constitutive SOCE in primary cultures of CRC cells is to refill the endogenous Ca^2+^ reservoir. Accordingly, the pharmacological and genetic blockade of resting Ca^2+^ influx prevents both CPA- and InsP_3_-induced ER Ca^2+^ release, thereby confirming that Stim- and Orai-mediated constitutive Ca^2+^ entry contributes to replenish the ER with Ca^2+^ in a SERCA-dependent manner. The same mechanisms has been recently unveiled in primary cultures of IH-ECFCs, which express a Stim1-, Orai1, and TRPC1-mediated constitutive SOCE [15]. Similar to CRC cells, the pharmacological inhibition of this resting Ca^2+^ entry with Pyr6 and 10 μM La^3+^ suppressed both CPA- and InsP_3_-induced ER Ca^2+^ release [15]. These findings provide the first evidence that Stim and Orai proteins mediate constitutive Ca^2+^ entry in CRC cells, whereas previous valuable studies were conducted on long term cancer cell lines and rather focused on agonist-induced SOCE [9, 50, 58, 59]. This feature could explain why resting Ca^2+^ entry has not described before in CRC [60]. Nevertheless, constitutive Orai1-dependent Ca^2+^ entry has been reported in both non-metastatic (MCF-7) and metastatic (MDA-MB-435) breast cancer cell lines [17, 18]. In breast cancer cells, however, Orai1 gating is independent on Stim1 and is not driven by ER Ca^2+^ store depletion; accordingly, Orai1 is activated by the Secretory Pathway Ca^2+^-ATPase, SPCA2, in MCF-7 cells [17] and by a yet to be identified mechanism in MDA-MB-435 cells [18].

Stim2 has long been regarded as the main determinant of resting Ca^2+^ entry and ER Ca^2+^ content [35, 61, 62]. Accordingly, Stim2 proteins displays a 2-fold lower affinity for Ca^2+^ as compared to Stim1 and is predicted to be activated already at the physiological ER Ca^2+^ concentration ([Ca^2+^]_ER_) (i.e. ≈500 μM) [35, 61, 62]. Nevertheless, it has been recently demonstrated that Stim2 recruits Stim1 to ER-plasma membrane junctions to gate Orai1 upon low ER Ca^2+^ depletion [63, 64]. Moreover, the ER Ca^2+^ store is largely depleted in CRC cells, as shown by the present investigation, as well as by previous reports [9, 60]. For instance, the passive ER Ca^2+^ leak induced by blocking SERCA activity with CPA leads to a small and localized elevation in [Ca^2+^]_i_ so that no global elevation in [Ca^2+^]_i_ occurs, as also described in RBL-1 cells stimulated with low doses of thapsigargin [65]. Under these conditions of large ER Ca^2+^ depletion, both Stim isoforms could be activated and gate Orai channels [61, 62], as reported in MCF-7 and MDA-MB-231 breast cancer cells [12, 66], LNCaP prostate cancer cells [33], murine B and T cells [67, 68], human myoblasts [69], and HeLa cells [70]. Stim1 and Stim2 may both interact with and gate Orai1 and Orai3 to mediate SOCE [5, 44, 61, 62]. Orai1 and Orai3 may associate into a heteromeric complex which can require [71] or not [20] Stim1 and is induced by AA or leukotriene C4 [72, 73]. However, we could not detect AA-induced Ca^2+^ entry in CRC cells. Nevertheless, a recent investigation clearly showed that heteromeric Orai1/Orai3 channels mediate Stim1- and Stim2-dependent SOCE induced by 5α-dihydrotestosterone (DHT) in LNCaP cells [33]. In addition, Stim1, Stim2, Orai1 and Orai3 were found to mediate SOCE in mouse dorsal root ganglion neurons [74] and mouse astrocytes [75]. Finally, Orai1 and Orai3 were shown to mediate Ca^2+^ entry in response to store depletion in mouse pulmonary artery smooth muscle cells [76]. Electrophysiological recordings revealed that heteromeric Orai1/Orai3 channels mediate a less Ca^2+^-selective SOCE in heterologous expression systems [77]. Further studies are required to understand whether Orai1 and Orai3 assemble into a heteromeric channel also in patients-derived CRC cells or act independently to mediate extracellular Ca^2+^ entry. Intriguingly, 2-APB caused a biphasic increase in [Ca^2+^]_i_ that was attenuated by the genetic deletion of Stim1, Orai1 and Orai3. This finding is somehow in line with the constitutive activation of SOCE in CRC cells: accordingly, 2-APB evoked a transient increase, followed by a slight reduction, in store-operated currents recorded from HEK 293 cells transfected with Stim1, Orai1 and Orai3 [77]. Nevertheless, there was no difference in the amplitude of 2-APB-evoked Ca^2+^ entry between pCRC and mCRC cells, although constitutive SOCE was up-regulated in pCRC cells. We should recall here that 50 μM 2-APB might directly activate also the remaining homomeric Orai3 channels and that Orai3 proteins are up-regulated in mCRC cells [77]. This feature could explain the lack of difference in the magnitude of the Ca^2+^ response to 2-APB between pCRC and mCRC cells.

Serum is a rich source of growth factors that may promote proliferation and migration by stimulating intracellular Ca^2+^ signals [41]. Previous study demonstrated that Stim1 and/or Orai1 mediated FBS-induced Ca^2+^ signals in the highly aggressive MDA-MB-231 human breast cancer cell line [78] and in the WM793 human melanoma cell line [79], thereby promoting tumor cell migration. Likewise, Stim1 and/or Orai1 supported cell proliferation [50, 59, 80] and migration [45, 58] in long term CRC cell lines. Herein, we showed that pharmacological blockade of constitutive SOCE or genetic silencing of Stim and Orai proteins prevented ER Ca^2+^ refilling and suppressed the Ca^2+^ response to FBS in primary cultures of CRC cells. However, FBS was still able to fully induce proliferation and migration also under these conditions. A recent study showed that Orai1 and Orai3 stimulated cell growth in Human Embryonic Kidney 293 cells, human hepatoma cells (Huh-7) and HeLa cells independently on their ion-conducting properties [81]. Functional assays revealed that mCRC cell proliferation and migration are not affected in Stim1-, Orai1- and Orai3-deficient cells, thereby confirming that Stim and Orai proteins do not mediate tumor growth and metastases in patients-derived CRC cells. As mentioned above, the pharmacological blockade of SOCE did not affect proliferation also in primary cultures of metastatic RCC cells [22] and of glioblastoma cells [23], although Stim1 and Orai1 promoted migration and invasion in the latter [23]. Therefore, the available evidence indicates that SOCE does not control proliferation in patients-derived cancer cells, while its role in migration may depend on the tumor type. This hypothesis is further supported by the observation that Pyr6 and La^3+^ block these processes in SW480 cells, which represent a widely employed model of metastatic CRC. However, we cannot rule out the possibility that Stim and Orai control alternative processes in primary cultures of CRC cells, such as resistance to apoptosis, cellular bioenergetics, insensitivity to antigrowth signals and adhesion to the substrate [3, 6, 8].

In conclusion, the present investigation provides the first evidence that Stim and Orai proteins mediate constitutive Ca^2+^ entry and control the ER Ca^2+^ content in primary cultures of human CRC cells. Constitutive SOCE does not drive CRC cell proliferation and migration, although this finding does not rule out the possibility that alternative Ca^2+^ entry/release pathways control CRC growth and metastasis in individuals affected by this malignancy. However, we confirmed that the pharmacological abrogation of SOCE inhibited proliferation and migration in immortalized CRC cells. These findings strongly suggest that the essential role of SOCE in cancer cell lines might overrate its actual contribution to cancer development and metastasis in human patients. This discrepancy is not surprising as it has long been known that immortalized tumor cell lines cannot recapitulate the complex biology of tumor microenviroment [6, 48, 82]. Similarly, VEGF has long been predicted to stimulate tumor vascularization and, therefore, has been put forward as an effective target for anti-angiogenic therapies [83, 84]. However, VEGF failed to stimulate pro-angiogenic intracellular Ca^2+^ signals in several types of cancer patients-derived endothelial progenitor cells [14, 15, 27, 85]. Therefore, caution is warranted when the Ca^2+^ toolkit is presented as a promising target for anticancer treatments only based on studies conducted on immortalized cancer cell lines [48, 86].

## MATERIALS AND METHODS

### Expansion of tumor cells

After signing an informed consent, patients (> 18 years) affected by mCRC who had undergone surgery intervention to remove primary tumor and/or liver metastases, were enrolled. All procedures were performed according to the guidelines prescribed for the treatment of CRC neoplasia, and no patient was subjected to unnecessary invasive procedures. Tumor specimens were processed as previously described, [25] with the GentleMACS Dissociator (Miltenyi Biotec, Germany) after being treated, with Tumor dissociation Kit (Miltenyi Biotec, Germany), according to the manufacturers’ instructions. Tumor cells were filtered to remove clusters, checked for viability with trypan blue die exclusion and resuspended at a concentration of 0.5-1x10^6^ cells/ml of CellGro SCGM (Cell Genix, Freiburg, Germany), supplemented with 20% FBS, 2mM L- glutamine, (complete medium) (Life Technologies Inc) and cultured in 25cm2 tissue flasks (Corning, Stone Staffordshire, England) at 37°C and 5% CO_2_. Viable tumor cells attached to the flask within 12-24 hours. Cultures at 75% to 100% confluence were selected for subculture by trypsinization with 0.25% trypsin and 0.02% EDTA (Life Technologies Inc) in a calcium/magnesium-free balanced solution. The culture medium was changed twice a week and cellular homogeneity evaluated microscopically every 24-48 hours. Cells were cryopreserved in 90% FBS and 10% DMSO and stored in liquid nitrogen for further experiments. To confirm the neoplastic origin of cultured cells obtained after 3-5 passages underwent to morphological and immunocytochemical analysis [25].

For proliferation assays, tumor cells were thawed and plated at the concentration of 10-20 x 10^5^/ml and evaluated after 3-4 days when reached the optimal confluency, as described in [22]. Results are expressed as average number of cells (± SE) under each condition; the cells were obtained from all four patients. Differences were assessed by the Student *t*-test for unpaired values. All statistical tests were carried out with GraphPad Prism 4.

### RNA isolation and real time RT-PCR (qRT-PCR)

Total RNA was extracted from the PCRC and MCRC using the QIAzol Lysis Reagent (QIAGEN, Italy). Single cDNA was synthesized from RNA (1 μg) using random hexamers and M-MLV Reverse Transcriptase (Promega, Italy). Reverse transcription was always performed in the presence or absence (negative control) of the reverse transcriptase enzyme. qRT-PCR was performed in triplicate using 1 μg cDNA and specific primers (intron-spanning primers) (Table 1). Briefly, GoTaq qPCR Mastermix (Promega, Italy) was used according to the manufacturer instruction and qRT-PCR performed using Rotor Gene 6000 (Corbett, Concorde, NSW, Australia). The conditions were as follows: initial denaturation at 95°C for 5 min; 40 cycles of denaturation at 95°C for 30 sec; annealing at 58°C for 30 sec, and elongation at 72°C for 40 sec. The qRT-PCR reactions were normalized using tyrosine 3-monooxygenase/tryptophan 5-monooxygenase activation protein, zeta polypeptide (Ywhatz), β-2-microglobulin (B2M), ubiquitin C (UBC) as housekeeping genes. The triplicate threshold cycles (Ct) values for each sample were averaged resulting in mean Ct values for both the gene of interest and the housekeeping genes. The gene Ct values were then normalized to the housekeeping gene by taking the difference: ΔCt = Ct[gene] - Ct[housekeeping], with high ΔCt values reflecting low mRNA expression levels. Melting curves were generated to detect the melting temperatures of specific products immediately after the PCR run. However, PCR products were also separated with agarose gel electrophoresis, stained with ethidium bromide. The molecular weight of the PCR products was compared to the DNA molecular weight marker VIII (Roche Molecular Biochemicals, Italy).

**Table 1:** Primer sequences used for real time reverse transcription/polymerase chain reaction

Gene	Primer sequences		Size (bp)	Accession number
Orai1	Forward	5′-AGTTACTCCGAGGTGATGAG-3′	257	NM_032790.3
	Reverse	5′-ATGCAGGTGCTGATCATGAG-3′		
Orai2	Forward	5′-CCATAAGGGCATGGATTACC-3′	334	NM_001126340.1 variant 1
	Reverse	5′-CAGGTTGTGGATGTTGCTCA-3′		NM_032831.2 variant 2
Orai3	Forward	5′-CCAAGCTCAAAGCTTCCAGCC-3′	159	NM_152288.2
	Reverse	5′-CAAAGAGGTGCACAGCCACCA-3′		
Stim1	Forward	5′-CCTCAGTATGAGGAGACCTT-3′	347	NM_003156.3
	Reverse	5′-TCCTGAAGGTCATGCAGACT-3′		
Stim2	Forward	5′-AAACACAGCCATCTGCACAG-3′	186	NM_020860.2
	Reverse	5′-GGGAAGTGTCGTTCCTTTGA-3′		
InsP_3_R1	Forward	5′-TCAACAAACTGCACCACGCT-3′	180	ENSG00000150995
	Reverse	5′-CTCTCATGGCATTCTTCTCC-3′		
InsP_3_R2	Forward	5′-ACCTTGGG GTTAGTGGATGA-3′	158	ENSG00000123104
	Reverse	5′-CCTTGTTTGGCTTGCTTTGC-3′		
InsP_3_R3	Forward	5′-TGGCTTCATCAGCACTTTGG-3′	173	ENSG00000096433
	Reverse	5′-TGTCCTGCTTAGTCTGCTTG-3′		
TRPC1	Forward	5′-ATCCTACACTGGTGGCAGAA-3′	307	NM_003304.4
	Reverse	5′-AACAAAGCAAAGCAGGTGCC-3′		
TRPC3	Forward	5′-GGAGATCTGGAATCAGCAGA-3′	336	NM_001130698.1 variant 1
	Reverse	5′-AAGCAGACCCAGGAAGATGA-3′		NM_003305.2 variant 2
TRPC4	Forward	5′-ACCTGGGACCTCTGCAAATA-3′	300	NM_016179.2 variant alpha
	Reverse	5′-ACATGGTGGCACCAACAAAC-3′		NM_001135955.1 variant beta
				NM_001135956.1 variant gamma
				NM_001135957.1 variant delta
				NM_003306.1 variant epsilon
				NM_001135958.1 variant zeta
TRPC5	Forward	5′-GAGATGACCACAGTGAAGAG-3′	221	NM_012471.2
	Reverse	5′-AGACAGCATGGGAAACAGGA-3′		
TRPC6	Forward	5′-AGCTGTTCCAGGGCCATAAA-3′	341	NM_004621.5
	Reverse	5′-AAGGAGTTCATAGCGGAGAC-3′		
TRPC7	Forward	5′-CACTTGTGGAACCTGCTAGA-3′	387	NM_020389.1
	Reverse	5′-CATCCCAATCATGAAGGCCA-3′		
Ywhatz^*^	Hs_YWHATZ_1_SG, QuantiTect Primer Assay QT00087962		71	NM_001135699
B2M^*^	Hs_B2M_1_SG QuantiTect Primer Assay QT00088935		98	NM_004048
UBC^*^	Hs_UBC_1_SG QuantiTect Primer Assay QT00234430		123	NM_021009

Tyrosine 3-monooxygenase/tryptophan 5-monooxygenase activation protein, zeta polypeptide (Ywhatz), β-2-microglobulin (B2M), Ubiquitin C (UBC); ^*^, from Qiagen.

### Membrane preparation and immunoblotting

Cells were homogenized by using a Dounce homogenizer in a solution containing: 250 mM Sucrose, 1 mM EDTA, 10 mM Tris-HCl, pH 7.6, 0.1 mg/ml PMSF, 100 mM β-mercaptoethanol, Protease and Phosphatase Inhibitor Cocktails (P8340 and P5726, P0044, Sigma-Aldrich Inc.). The homogenates were solubilized in Laemmli buffer [87] and 30 μg proteins were separated on 12% SDS-polyacrilamide gel electrophoresis and transferred to the Hybond-P PVDF Membrane (GE Healthcare, Italy) by electroelution. After 1 h blocking with Tris buffered saline (TBS) containing 3% BSA and 0.1% Tween (blocking solution) the membranes were incubated overnight at 4°C with the following antibodies diluted in the TBS and 0.1% Tween: anti-Stim1 (sc-166840; 1:350, dilution), anti-Orai1 (sc-68895; 1:200, dilution) anti-InsP_3_R-1/2/3 (sc-377518; 1:200, dilution) from Santa Cruz Biotechnology (CA, USA), anti-Stim2 (PRS4123; 1:500, dilution), anti-Orai3 (HPA015022; 1:500, dilution) from Sigma-Aldrich (Italy). The membranes were washed and incubated for 1 h with peroxidase-conjugated goat anti-rabbit IgG (Chemicon, AP132P) or peroxidase-conjugated rabbit anti-mouse IgG (Dakocytomation, P0260), diluted 1:120000 in blocking solution. The bands were detected with ECL™ Select western blotting detection system (GE Healthcare Europe GmbH, Italy). Prestained molecular weight markers (ab116028, Abcam) were used to estimate the molecular weight of the bands. Blots were stripped as shown in [22, 26] and re-probed with with RabMAb anti β-2-microglobulin antibody ([EP2978Y] ab75853; ABCAM) as housekeeping. The antibody was diluted 1:10000 in blocking solution.

### Protein content

Protein contents of all the samples were determined by the Bradford’s (Bradford, 1976) method using bovine serum albumin (BSA) as standard.

### Gene silencing

siRNA targeting Stim1, Orai1, Orai3 were purchased by Sigma-Aldrich Inc. MISSION esiRNA (hStim1, EHU026401; hOrai1, EHU120081; hOrai3, EHU131741). Scrambled siRNA were used as negative control. Briefly, once the monolayer cells had reached 50% confluency, the medium was removed and the cells were added with Opti-MEM I reduced serum medium without antibiotics (Life technologies, U.S.A.). siRNAs (100 nM final concentration) were diluted in Opti-MEM I reduced serum medium and mixed with Lipofectamine™ RNAiMAX transfection reagent (Invitrogen) pre-diluted in Opti-MEM), according to the manufacturer’s instructions. After 20 min incubation at room temperature, the mixes were added to the cells and incubated at 37°C for 5 h. Transfection mixes were then completely removed and fresh culture media was added. The effectiveness of silencing was determined by immunoblotting (see Figure 10) and silenced cells were used 24 h or 48 h after transfection for PCRC and MCRC, respectively.

### Solutions for intracellular Ca^2+^ recordings

Physiological salt solution (PSS) had the following composition (in mM): 150 NaCl, 6 KCl, 1.5 CaCl2, 1 MgCl2, 10 Glucose, 10 Hepes. In Ca^2+^-free solution (0Ca^2+^), Ca^2+^ was substituted with 2 mM NaCl, and 0.5 mM EGTA was added. Solutions were titrated to pH 7.4 with NaOH. In Mn^2+^-quenching experiments, 200 mM MnCl_2_ was added to the 0Ca^2+^ external solution. The osmolality of PSS as measured with an osmometer (Wescor 5500, Logan, UT) was 338 mmol/kg.

### [Ca^2+^]_i_ measurements

CRC cells were loaded with 4 μM fura-2 acetoxymethyl ester (fura-2/AM; 1 mM stock in dimethyl sulfoxide) in PSS for 1 hour at room temperature. After washing in PSS, the coverslip was fixed to the bottom of a Petri dish and the cells observed by an upright epifluorescence Axiolab microscope (Carl Zeiss, Oberkochen, Germany), usually equipped with a Zeiss ×40 Achroplan objective (water-immersion, 2.0 mm working distance, 0.9 numerical aperture). ECFCs were excited alternately at 340 and 380 nm, and the emitted light was detected at 510 nm. A first neutral density filter (1 or 0.3 optical density) reduced the overall intensity of the excitation light and a second neutral density filter (optical density=0.3) was coupled to the 380 nm filter to approach the intensity of the 340 nm light. A round diaphragm was used to increase the contrast. The excitation filters were mounted on a filter wheel (Lambda 10, Sutter Instrument, Novato, CA, USA). Custom software, working in the LINUX environment, was used to drive the camera (Extended-ISIS Camera, Photonic Science, Millham, UK) and the filter wheel, and to measure and plot on-line the fluorescence from 10 up to 40 rectangular “regions of interest” (ROI). Each ROI was identified by a number. Since cell borders were not clearly identifiable, a ROI may not include the whole cell or may include part of an adjacent cell. Adjacent ROIs never superimposed. [Ca^2+^]_i_ was monitored by measuring, for each ROI, the ratio of the mean fluorescence emitted at 510 nm when exciting alternatively at 340 and 380 nm (shortly termed “ratio”). An increase in [Ca^2+^]_i_ causes an increase in the ratio [30, 88]. Ratio measurements were performed and plotted on-line every 3 s. The experiments were performed at room temperature (22°C).

### Cell migration assay

Cell migration assay was performed with the scratch wound healing analysis. Briefly, cells were seeded in 12-well plates to form a confluent pCRC/mCRC monolayer and were maintained at 37°C with 5% CO_2_ incubator until confluence was reached. After removing culture medium, a couple of washing with 1 mL PBS have been performed and then the monolayer was scratched by using a sterile 0.1 - 10 μL pipette tip to create a wound-like gap; floating cells were removed by washing another time in PBS solution and finally cells were treated with experimental conditions. The assay was performed on pCRC and mCRC monolayers exposed to the culture medium alone, as a control, and on pCRC and mCRC monolayers grown in the presence of the standard cultre medium supplemented with Pyr6 (10 μM, 30 min), La^3+^ (10 μM, 30 min) or G418 (100 μM, 48 h)as well as on siStim1, siOrai1, siOrai3 mCRC monolayers grown to confluence with the culture medium. Cells were left to migrate for 24 hours and the width of the wound space was measured at wounding and at the end of treatment, by using microscope equipped with a camera. Measurements of wound width were made for each wound at randomly chosen points. Wound closure rates were determined as the difference between wound width at 0 and 48 hours. All the experiments were carried out on pCRC and mCRC cells isolated from 4 different donors (when possible, pCRC and mCRC cells were deriving from the same donor). As a control for immortalized CRC cells, the effect of Pyr6 (10 μM, 30 min) and La^3+^ (10 μM, 30 min) was assessed on SW480 cells (obtained from ATCC). SW480 cells were grown in RPMI 1640 supplemented with 10% FCS.

### Statistics

All the Ca^2+^ data have been collected from at least 4 different pCRC and mCRC cells harvested from different patients (when possible, pCRC and mCRC cells were deriving from the same donor). Pooled data are given as mean±SE and statistical significance (P<0.05) was evaluated by the Student’s *t*-test for unpaired observations. The amplitude of Ca^2+^ release in response to either CPA or ATP was measured as the difference between the ratio at the peak of intracellular Ca^2+^ mobilization and the mean ratio of 1 min baseline before the peak. The magnitude of SOCE evoked by either CPA or ATP upon Ca^2+^ restoration to the bath was measured as the difference between the ration at the peak of extracellular Ca^2+^ entry and the mean ration of 1 in baseline before Ca^2+^ readdition. The rate of Mn^2+^ influx was evaluated by measuring the slope of the fluorescence intensity curve at 400 sec after Mn^2+^ addition [89].

As to mRNA and protein analysis, all data are expressed as mean ± SE. The significance of the differences of the means was evaluated with Student’s *t* test. In the proliferation assays, results are expressed as percentage (± SE) of growth compared to controls (given as 100% growth), obtained from 4 different sets of experiments, each performed in duplicate. Differences were assessed by the Student *t*-test for unpaired values. All statistical tests were carried out with GraphPad Prism 4.

As to cell migration assays, the effect of the pharmacological or genetic treatments was evaluated as the percentage of cell migration inhibition under the designated treatments. Statistical analysis was carried out with GraphPad Prism 4.

## SUPPLEMENTARY MATERIALS FIGURES


