# Supplementary material for: Stim and Orai mediate constitutive Ca2+ entry and control endoplasmic reticulum Ca2+ refilling in primary cultures of colorectal carcinoma cells
Source: Oncotarget. 2018 Jul 24;9(57):31098–119. doi: 10.18632/oncotarget.25785 (PMC6089563; doi:10.18632/oncotarget.25785)
Supplement: Supplementary file 1 [file oncotarget-09-31098-s001.pdf]

Stim and Orai mediate constitutive Ca^{2+} entry and control endoplasmic reticulum Ca^{2+} refilling in primary cultures of colorectal carcinoma cells

SUPPLEMENTARY MATERIALS

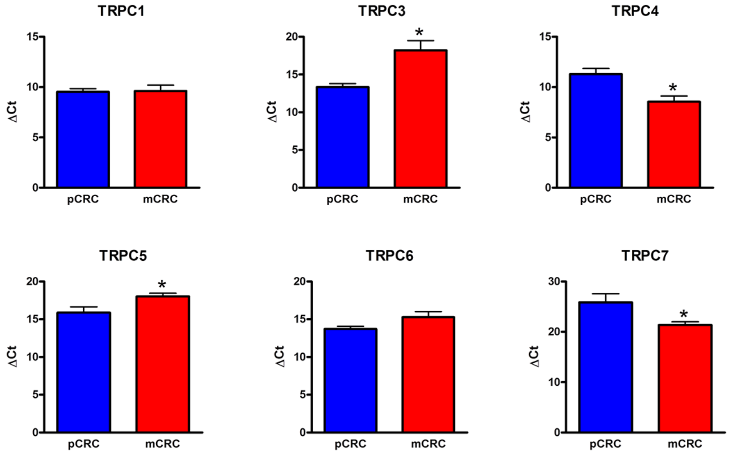

Supplementary Figure 1: Expression of TRPC channel transcripts in patients-derived colorectal cancer cells. Quantitative real-time reverse transcription polymerase chain reaction of total RNA was performed using specific primers as indicated in Table 1. The relative mRNA levels were determined as described in Materials and Methods. In each experiment the Ct values obtained were normalized to the Ct of three housekeeping genes and then averaged. Bars represent the mean±SEM of at least 4 different RNA extracts each from different patients. The asterisk indicates $p<0.05$ versus pCRC (Student's *t* test).

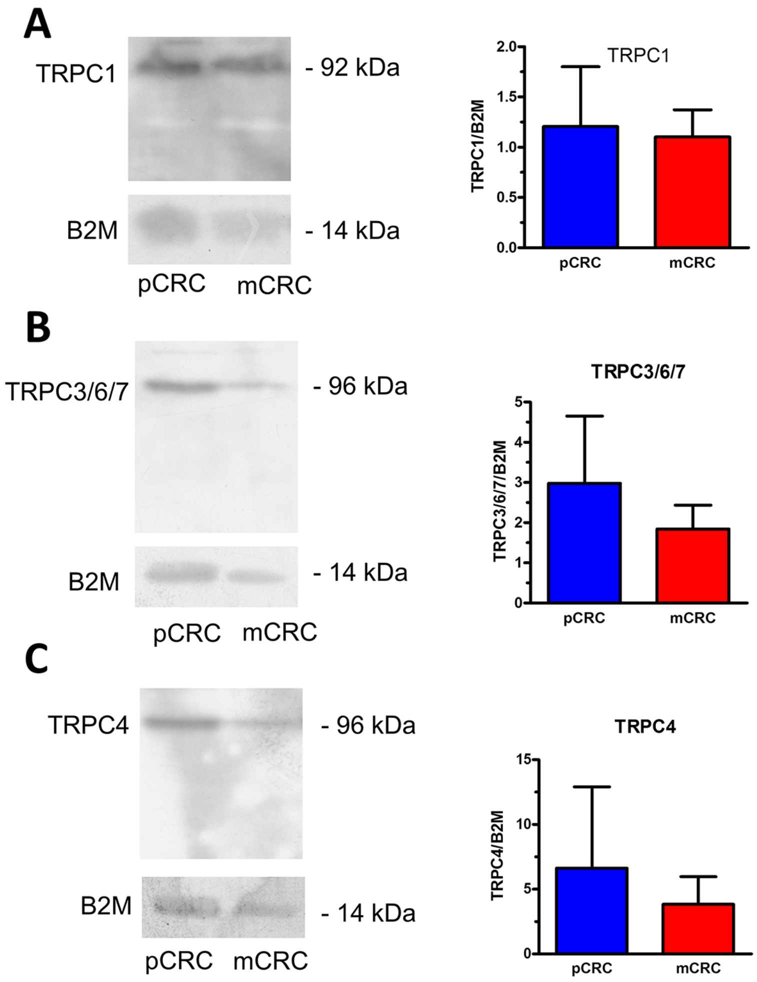

Supplementary Figure 2: Expression of TRPC channel proteins in patients-derived colorectal cancer cells. Blots representative of four (each from a distinct patient) were shown. Lanes were loaded with 30 µg of proteins, probed with affinity purified antibodies and processed as described in Materials and Methods. The same blots were stripped and re-probed with anti-beta-2-microglobulin (B2M) polyclonal antibody, as housekeeping. Major bands of the expected molecular weights were observed. Bands were acquired, densitometric analysis of the bands was performed by Total Lab V 1.11 computer program (Amersham Biosciences Europe, Italy) and the results were normalized to the corresponding B2M.

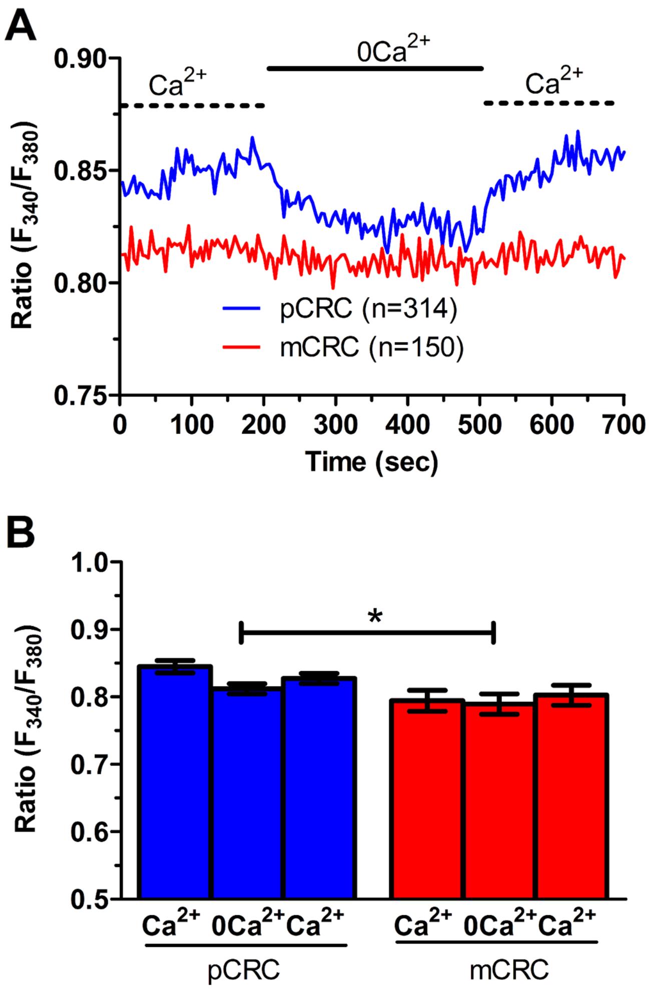

Supplementary Figure 3: Dependence of $[Ca^{2+}]_i$ on extracellular Ca^{2+} in patients-derived colorectal cancer cells. (A) perfusion with a recording solution devoid of Ca^{2+} ($0Ca^{2+}$) caused a rapid, reversible decrease in $[Ca^{2+}]_i$ in pCRC cells, but not in mCRC cells. **(B)** mean±SE of the reversible drop in $[Ca^{2+}]_i$ recorded in pCRC and mCRC cells caused by $0Ca^{2+}$ perfusion.

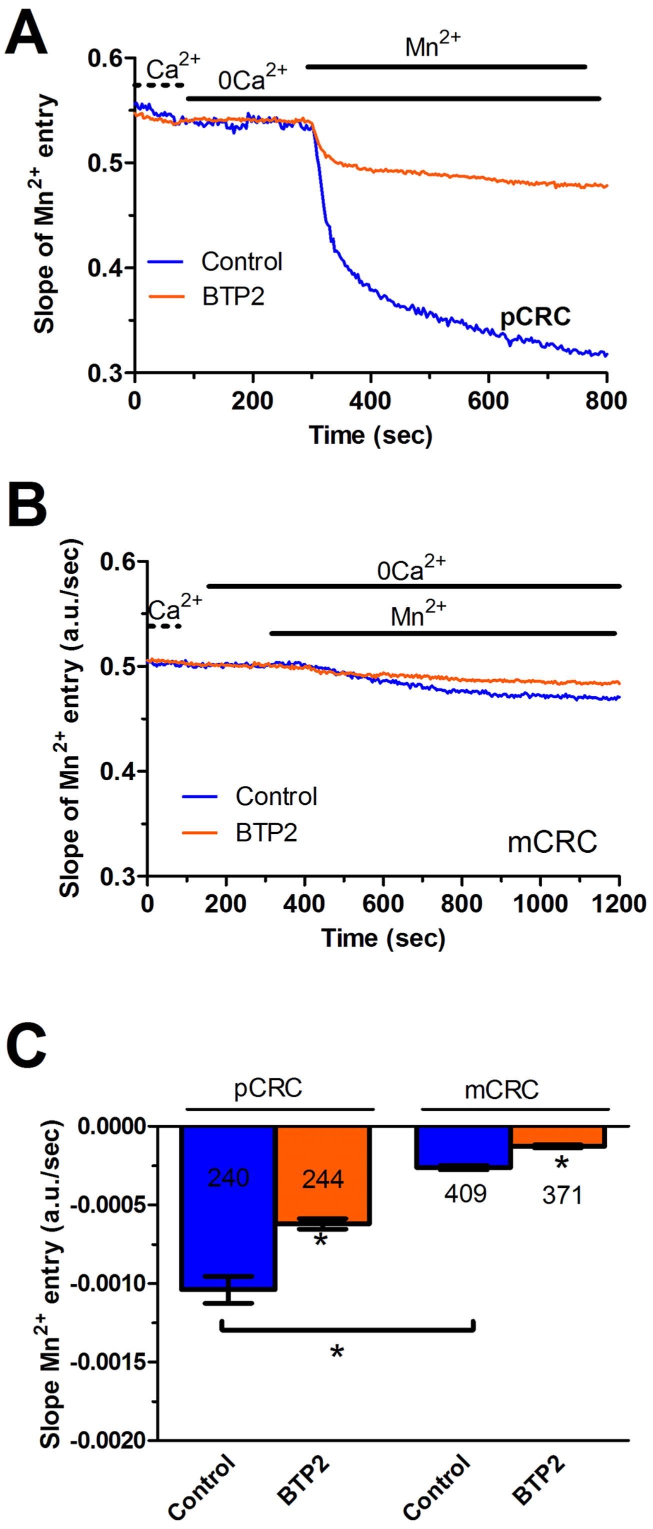

Supplementary Figure 4: YM-58483/BTP2 reduced the rate of extracellular Ca^{2+} entry in patients-derived colorectal cancer cells. **(A)** The rate of Mn^{2+} entry in pCRC cells was remarkably reduced upon pre-treatment with YM-58483/BTP2 (10 µM, 30 min), a widely employed Orai inhibitors. **(B)** The rate of Mn^{2+} entry in mCRC cells was remarkably reduced upon pre-treatment with YM-58483/BTP2 (10 µM, 30 min), a widely employed Orai inhibitors. **(C)** mean±SE of the quenching rate of Fura-2 fluorescence signal measured in pCRC and mCRC cells in the absence and in the presence of YM-58483/BTP2. The asterisk indicates $p<0.05$.

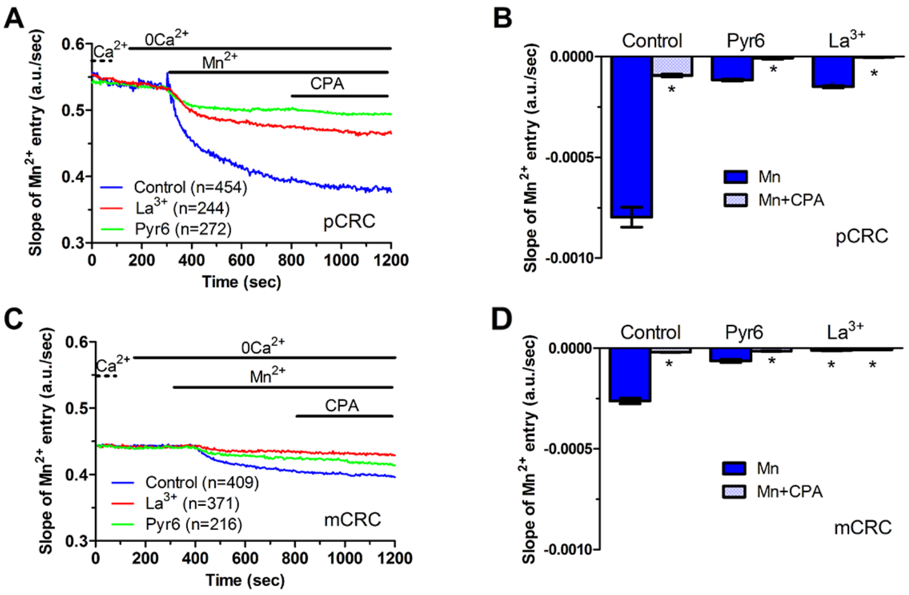

Supplementary Figure 5: CPA enhanced the rate of extracellular Ca^{2+} entry in patients-derived colorectal cancer cells. (A) CPA (10 µM) caused a clear increase in the rate of Mn^{2+} entry in pCRC cells, which was remarkably reduced upon pre-treatment with either Pyr6 (10 µM, 30 min) or La^{3+} (10 µM, 30 min). **(B)** mean±SE of the quenching rate of Fura-2 fluorescence signal measured in pCRC cells before and after CPA addition and after CPA addition in the absence and in the presence of Pyr6 and La^{3+}. The asterisk indicates $p<0.05$. **(C)** CPA (10 µM) caused a clear increase in the rate of Mn^{2+} entry in mCRC cells, which was remarkably reduced upon pre-treatment with either Pyr6 (10 µM, 30 min) or La^{3+} (10 µM, 30 min). **(D)** mean±SE of the quenching rate of Fura-2 fluorescence signal measured in mCRC cells before and after CPA addition and after CPA addition in the absence and in the presence of Pyr6 and La^{3+}. The asterisk indicates $p<0.05$.

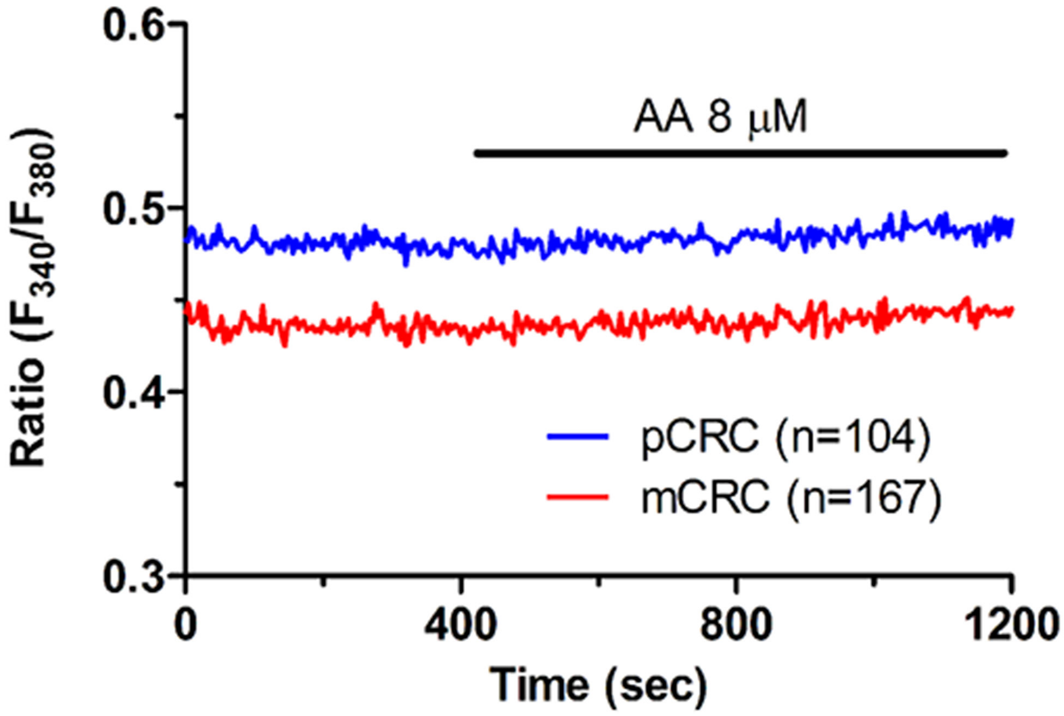

Supplementary Figure 6: Arachidonic acid does not elicit any detectable increase in $[Ca^{2+}]_i$ in patients-derived colorectal cancer cells. Arachidonic acid (AA; 8 µM) caused no detectable in $[Ca^{2+}]_i$ in mCRC and pCRC cells. The baseline of the Ca^{2+} traces was adjusted for representative purposes.

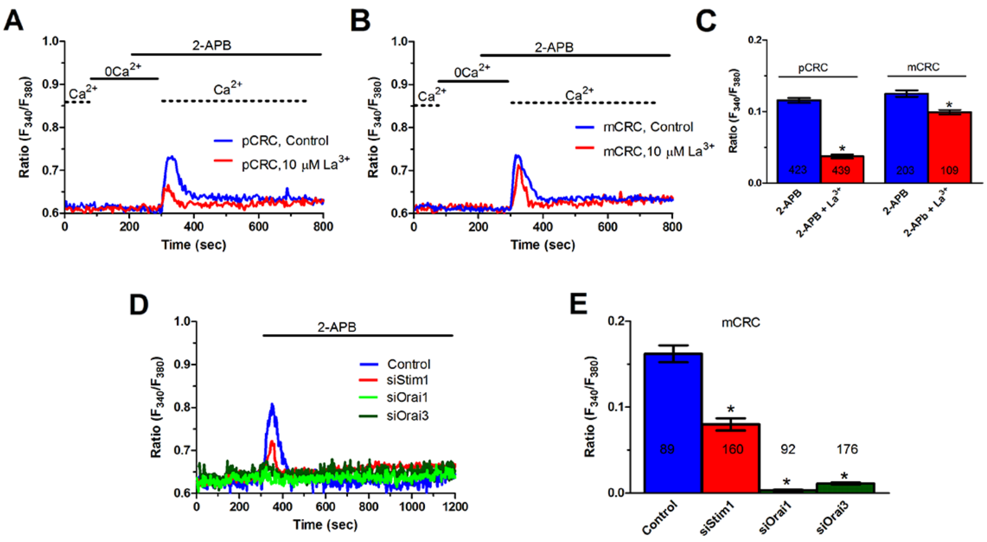

Supplementary Figure 7: 2-APB-evoked extracellular Ca^{2+} entry in patients-derived colorectal cancer cells. **(A)** 2-APB (50 μM) caused an increase in $[Ca^{2+}]_i$ in pCRC cells only in the presence, but not in the absence (0 Ca^{2+}), of extracellular Ca^{2+}. 2-APB-evoked Ca^{2+} entry was reduced by pre-treating the cells with La^{3+} (10 μM, 30 min). **(B)** 2-APB (50 μM) caused an increase in $[Ca^{2+}]_i$ in mCRC cells only in the presence, but not in the absence (0 Ca^{2+}), of extracellular Ca^{2+}. 2-APB-evoked Ca^{2+} entry was reduced by pre-treating the cells with La^{3+} (10 μM, 30 min). **(C)** mean±SE of the amplitude of 2-APB-evoked Ca^{2+} entry in pCRC and mCRC cells in the absence and in the presence of La^{3+}. **(D)** genetic silencing of Stim1, Orai1 and Orai3 strongly reduced extracellular Ca^{2+} entry induced by 2-APB (50 μM) in mCRC cells. **(E)** mean±SE of the amplitude of 2-APB-evoked Ca^{2+} entry in mCRC cells under the designated treatments.

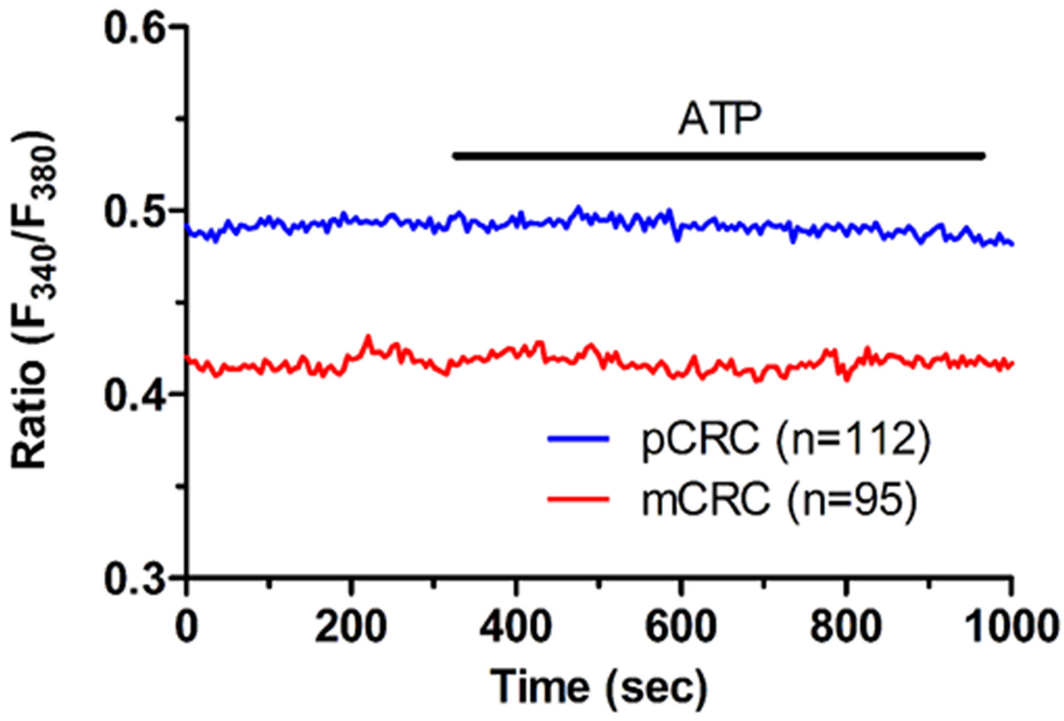

Supplementary Figure 8: ATP does not elicit any detectable increase in $[Ca^{2+}]_i$ in patients-derived colorectal cancer cells. ATP (100 μM) caused no detectable increase in $[Ca^{2+}]_i$ in pCRC and mCRC cells. The baseline of the Ca^{2+} traces was adjusted for representative purposes.

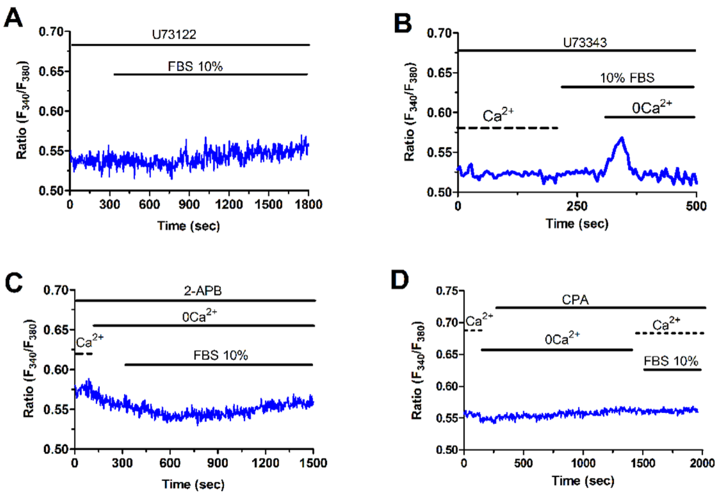

Supplementary Figure 9: FBS-induced intracellular Ca^{2+} release is mediated by $InsP_3Rs$ in primary colorectal cancer cells. 10% FBS-induced intracellular Ca^{2+} release under $0Ca^{2+}$ conditions ($0Ca^{2+}$) was inhibited by U73122 (10 µM, 30 min; n=123), an established PLC inhibitor **(A)** but it was not affected by its structurally inactive analogue, U73343 (10 µM; 30 min; n=167) **(B)** in pCRC cells. **(C)** 10% FBS-induced intracellular Ca^{2+} release under $0Ca^{2+}$ conditions was suppressed by 2-APB (50 µM, 30 min; n=102), which blocks $InsP_3Rs$, in pCRC cells. **(D)** depletion of the ER Ca^{2+} store with CPA (10 µM; n=134) under $0Ca^{2+}$ conditions prevented the subsequent FBS-induced intracellular Ca^{2+} release in pCRC cells.

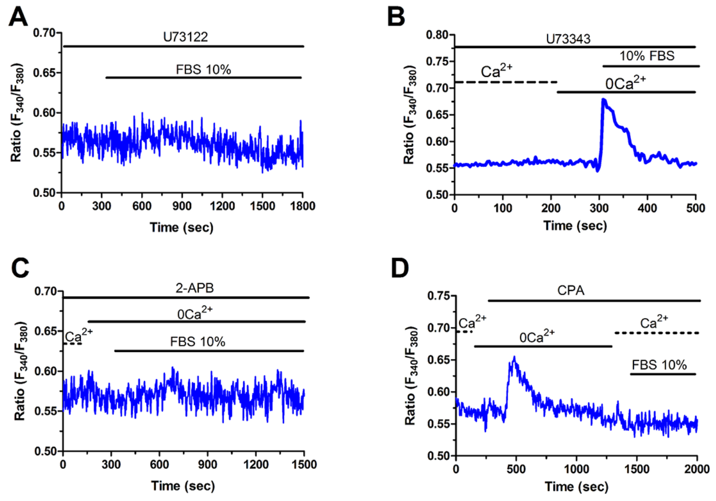

Supplementary Figure 10: FBS-induced intracellular Ca^{2+} release is mediated by $InsP_3Rs$ in metastatic colorectal cancer cells. 10% FBS-induced intracellular Ca^{2+} release under $0Ca^{2+}$ conditions ($0Ca^{2+}$) was inhibited by U73122 (10 µM, 30 min; n=112), an established PLC inhibitor **(A)** but it was not affected by its structurally inactive analogue, U73343 (10 µM; 30 min; n=105) **(B)** in mCRC cells. **(C)** 10% FBS-induced intracellular Ca^{2+} release under $0Ca^{2+}$ conditions was suppressed by 2-APB (50 µM, 30 min; n=113), which blocks $InsP_3Rs$, in mCRC cells. **(D)** depletion of the ER Ca^{2+} store with CPA (10 µM; n=126) under $0Ca^{2+}$ conditions prevented the subsequent FBS-induced intracellular Ca^{2+} release in mCRC cells.

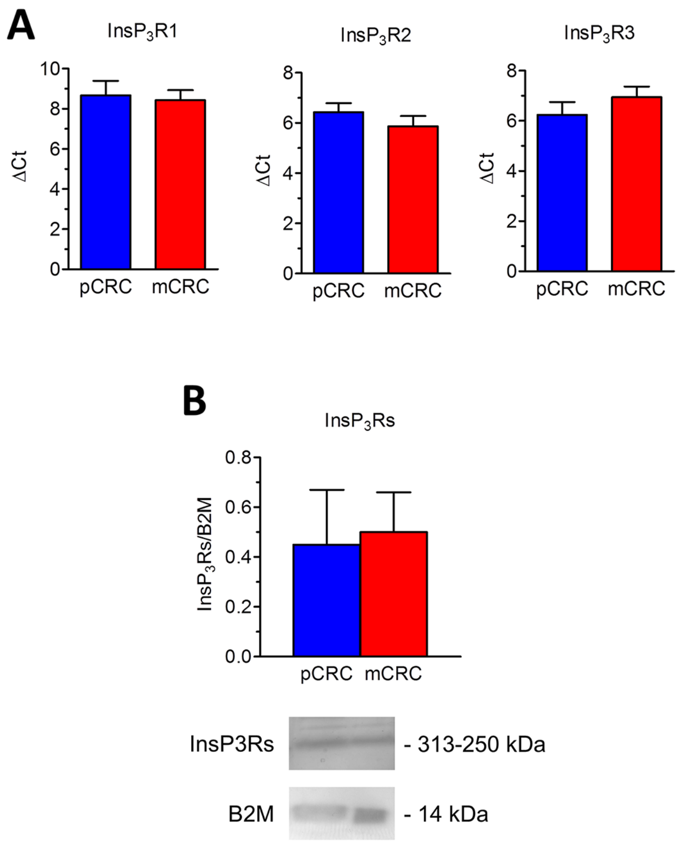

Supplementary Figure 11: $InsP_3Rs$ are not differentially expressed between primary and metastatic colorectal cancer cells. **(A)** Expression of $InsP_3R1$-3 transcripts in pCRC and mCRC cells. Quantitative real-time reverse transcription polymerase chain reaction of total RNA was performed using specific primers as indicated in Table 1. The relative mRNA levels were determined as described in Materials and Methods. In each experiment the Ct values obtained were normalized to the Ct of three housekeeping genes and then averaged. Bars represent the mean±SEM of at least 4 different experiments each from different RNA extracts. $^{*}P<0.05$ versus pCRC (Student's *t* test). **(B)** expression of $InsP_3R1$-3 proteins in pCRC and mCRC cells. Blots representative of four were shown. Lanes were loaded with 30 µg of proteins, probed with affinity purified antibodies and processed as described in Materials and Methods. The same blots were stripped and re-probed with anti-beta-2-microglobulin (B2M) polyclonal antibody, as housekeeping. Major bands of the expected molecular weights were observed. Bands were acquired, densitometric analysis of the bands was performed by Total Lab V 1.11 computer program (Amersham Biosciences Europe, Italy) and the results were normalized to the corresponding B2M.

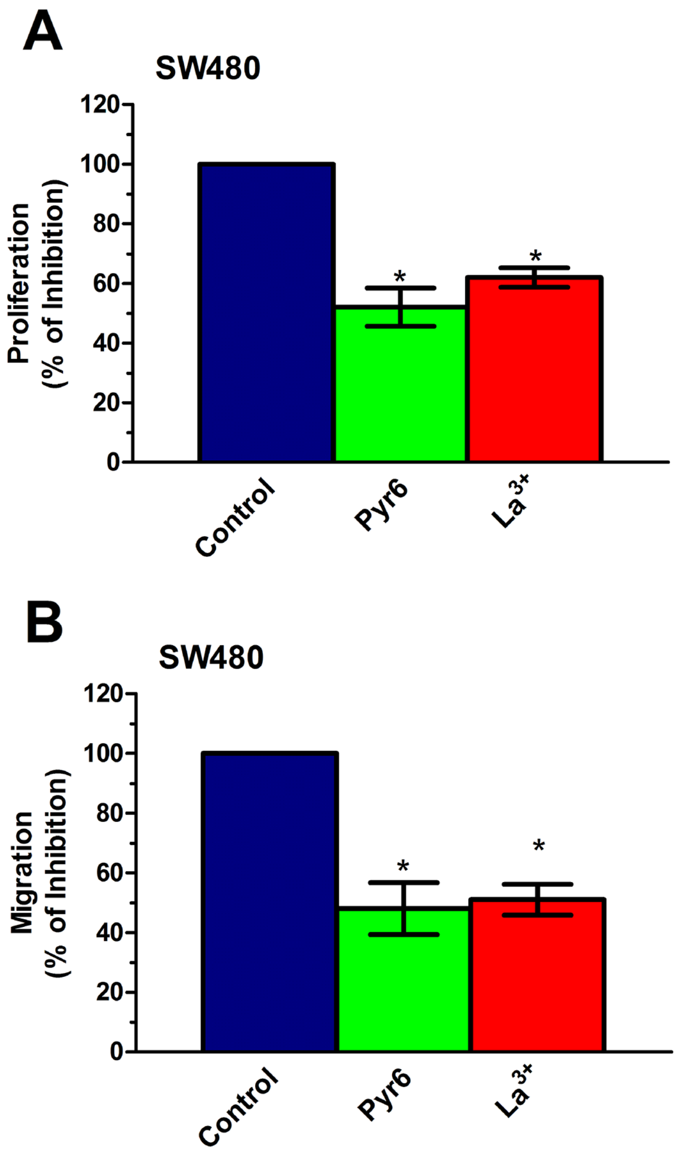

Supplementary Figure 12: Pharmacological inhibition of store-operated Ca^{2+} entry inhibits proliferation and migration in SW480 cells. Pre-incubating SW480 cells with Pyr6 (10 µM) or La^{3+} (10 µM) significantly reduced both proliferation **(A)** and migration **(B)**. The asterisk indicates $p<0.05$.
